# National pride and tax compliance: A laboratory experiment using a physiological marker

**DOI:** 10.1371/journal.pone.0280473

**Published:** 2023-01-19

**Authors:** Alison Macintyre, Ho Fai Chan, Markus Schaffner, Benno Torgler

**Affiliations:** 1 Centre for Behavioural Economics, Society & Technology (BEST), Queensland University of Technology, Brisbane, Australia; 2 School of Economics and Finance, Queensland University of Technology, Brisbane, Australia; 3 CREMA—Center for Research in Economics, Management, and the Arts, Basel/Zürich, Switzerland; University of Crete: Panepistemio Kretes, GREECE

## Abstract

This paper reports on a laboratory experiment designed specifically to test the influence of national pride on tax honesty while using a physiological marker to observe emotional responses to patriotic priming. Participants were exposed to one of three framing videos before earning income in a real effort task and were given the chance to declare their taxable income. We find that psychological priming through exposure to symbols of Australian national pride and national identity had a positive effect on the level of tax compliance among Australian but not non-Australians. In addition, non-Australians report lower tax compliance ratios in the treatment groups than in the control group which may indicate an outgroup effect. When exploring the potential of a physiological marker of national pride we observe two different types of physiological responses to the activation and effects of national pride and its impact on tax compliance among Australians. Iconic images activate the parasympathetic nervous system while sports scenes activate the sympathetic nervous system, but both types of images and responses are positively associated with tax compliance. In addition, we find that non-Australians resident in the country for more than a year report a higher level of tax compliance, and that there are some similarities in heart rate variability (HRV) responses between Australian citizens born in the country and those born overseas who have been in Australia for a longer period. Overall, the results support the proposition that identifying with an ingroup at a national level is important for tax compliance.

## 1. Introduction

The late (and great) Kenneth Boulding [1, p 93] stated that “the dynamics which govern the creation, destruction, and distribution of various forms of pride and shame in society are very little understood, yet nothing perhaps is more crucial to the understanding of the overall dynamics of a particular society than the marked differences which exist among societies in this regard”. As an important form of pride in societies across the world, the underlying dynamics of national pride suggest a particularly interesting topic of inquiry. The connection between ingroup identity and national pride raises questions about its potential to influence cooperation, and therefore what impact it might have on citizens’ willingness to pay taxes. Given that a well-organised tax regime is essential to the functioning of civilised society, an understanding of the overall dynamics between national pride and tax compliance is of special interest.

National pride can be defined as “the positive affect that the public feels towards their country as a result of their national identity. It is both the pride or sense of esteem that a person has for one’s nation and the pride or self-esteem that a person derives from one’s national identity” [2, p 1]. Of course, the idea that nationalistic propaganda is effective in prompting certain behavioural outcomes is not new; both modern and ancient history can testify to the results of overly-enthusiastic patriotic sentiment. In almost all countries, national pride is commonplace and employed as moral suasion across a range of contexts. Governments, businesses, and advertisers clearly believe that this sentiment can change economic decisions; actively appealing to the ideals of national pride and duty to secure solidarity and compliance among citizens. We know that governments all over the world expend significant resources in celebration of their national days, funding advertising campaigns, community fun-days, and fireworks. Girling [3, p 180] points out something that “American presidents, Democrats as well as Republicans, do as a matter of course. My fellow Americans. It harks to the founding principles of the Bill of Rights. To declare yourself American is to buy into an ideal. So it is to declare your yourself French: liberté, egalité, fraternité”.

We constantly absorb advertising messages and socialisation through activities and lessons at school [4]. The compulsory singing of the national anthem at school has even been defended by some scholars. Dr Kevin Donnelly–a senior research fellow at the Australian Catholic University and past chair of a review of the Australian national curriculum–declared in an opinion piece published in the *Sydney Morning Herald* that “if Australia is to be a cohesive, open and prosperous society instead of one where disunity prevails, future citizens must be taught to acknowledge and celebrate our national institutions and what makes us unique” [5]. Yet, it is reasonable to question the purpose behind creating and reinforcing this sense of belonging, pride, and celebration about being a citizen of a particular country. After all, nationality is a designation often based on the arbitrary geography of birth; it usually cannot in any way be characterised as a personal achievement; and could be regarded merely as a type of label. But daily exposure to national symbols [6], and exposure to the national discourse provides the context through which national pride is activated, and through which an individual might acquire an understanding of the characteristics of what is assumed to be a “good citizen”. For example, popular discourse regularly includes pronouncements on whether a behaviour is or is not ‘Australian’, ‘American’, or ‘British’, and so on. Both political rhetoric and commercial advertising appeal to national pride and patriotism in a range of situations, including the rationale behind pursuing a protectionist regime [7]; for encouraging the purchase of war bonds [8]; for investing money in sporting events (see Van Hilvoorde et al. [9], although the authors find no empirical evidence of the planned increase in national pride to support the political rhetoric); for advertising during sporting events [10]; for defence spending; to market particular products as ‘Made in the USA’, or ‘Proudly Australian’, and for maintaining strong economic growth through interventions designed to benefit only the nation state (as noted by Clift and Woll [11, p 308], the term “economic patriotism” was coined by the French Prime Minister). Citizens are encouraged to consume for the good of the country [12]; to have children for the good of the country [13]; and Newt Gingrich infamously claimed he loved his country so much it caused his infidelity to his wife [14].

Given that such a phenomenon is so prevalent in society, it would seem important to understand the implications; yet there is very little research available into the effect of being exposed to such symbols and messages in the context of compliance and cooperation. Slemrod [8, p 40] refers to the role of conscience in tax compliance, noting that “[a]ppeals to patriotism to induce citizens to pay their taxes (and, often, buy war bonds) are common in recent times”. However, he also stresses concerns regarding the efficacy of suasion on tax compliance: “That such campaigns are successful during ordinary (non-war) times in swaying taxpayers from their otherwise optimal compliance strategy has not been compellingly demonstrated” [8, p 40].

Previous empirical evidence has relied heavily on results from survey data, finding support for the proposition that national pride is correlated with a higher willingness to pay taxes [4, 15–17]. Wenzel [18] investigated three levels of taxpayers’ identity (at the personal, subgroup, and national level) and finds that an identity constructed at the national level is the most conducive for tax compliance, even going so far as to conclude that “the concept of identity is key to responsive regulation” [19, p 31]. Gangl et al. [19] report results of one survey and three experiments that manipulate patriotism by displaying either a national flag, national landscapes, or by priming national achievements. The findings indicate that emphasising national achievements promotes patriotism and increases cooperation and trust in public institutions, while focusing on national achievements or national landscapes increases citizens’ identification and cooperation with their community. Therefore, in this study we narrow the focus to specifically test the potential influence of national pride on tax compliance. We do this by using an experimental setting with a psychological priming event, combined with recording of physiological data that indicate whether the participants were relaxed or excited by the psychological trigger. Previous studies have introduced physiological measurements in the context of tax compliance [20] or social dilemmas [21]. Thus, we employ a standard tax compliance experiment (see [22–25]), adding to the protocol a psychological framing device designed to provoke a feeling of national pride.

Both Australian citizens and foreign nationals were exposed to one of three videos: the first depicting typically Australian iconic images, the second comprising footage of sporting achievements, and the third being a control video with randomised patterns. We also recorded the heart rate variability of the participants during the psychological priming and during their tax payment decisions. The results indicate that Australian citizens who are affected by the national pride priming exhibit a higher level of tax compliance (more likely to pay their taxes), and that there is also a difference in tax honesty between Australian and non-Australian participants. This is the novel result of the paper: we can control for the physiological effect of the national pride triggers, observing increased tax compliance in those who respond to the treatments.

## 2. Methods

### 2.1. Experimental design

The experiments were conducted over nine sessions at the Queensland Behavioural Economics Laboratory (henceforth referred to as QuBE), in a dedicated computer lab featuring partitions between the students to prevent copying or communication during the experiment. Adjoining the lab room (separated by a door) is a small office where the main terminal operated as the server from which the experiment was initiated and monitored. The experiments were carried out in line with the Queensland University of Technology (QUT) ethics requirements for research involving human participants, under the approval of QuBE and QUT’s Human Research Ethics Committee (QUT Ethics Approval Number 1300000142). The participants all gave informed consent after being made aware that it was an incentivised experiment and that physical measurements would be taken. The experiments were conducted during July and August 2013. Participants were recruited with ORSEE [26] from QUT’s standard participant pool for economic experiments.

From the subject group of 138 participants, 56 percent were male and 44 percent female. The sample comprised both international and domestic students, with 79 students not born in Australia and 59 students born in Australia. However, while 60 percent of students were born overseas, many have since become citizens, which indicates that they have been resident in the country for a considerable length of time. Of the foreign-born students who answered the question on whether they were a citizen, 28 percent were not, and 72 percent had become Australian citizens. A dummy variable was constructed to measure the citizenship status of the participants (Australia dummy), where 1 = all participants born in Australia, as well as those born overseas who have since become citizens; and 0 = non-citizens. The addition of students not born in Australia but who have since become citizens changed the proportion to 57% Australian citizens and 43% non-citizens. It can be presumed that those in the Australian citizen group were subjected to similar socialisation processes at school (singing the Australian national anthem) and at home (attending national celebration days such as Australia Day); would have observed the same pro-Australian messages in television advertisements; and were exposed to the same political rhetoric regarding what is ‘Australian’ and what is not. Students who had emigrated at a younger age would have been exposed to all these factors since residing in Australia, and those emigrating more recently would have at least partial exposure to rhetoric and socialisation (the process for categorising according to length of stay in Australia is discussed in section 3.4). The subject pool had fully randomised assignment into control and treatment group; that is, treatment assignment did not depend on the participant’s citizenship status, nor on other characteristics. Each participant was randomly assigned into a group of around 16 students and that group was randomly assigned to an experimental session.

The experiment lasted just under one hour. Students earned on average $19.33 in accumulated income (referred to as “wealth”) during the experiment, with a minimum earn of $10.15 and maximum of $29.19. All payments were made in Australian dollars (AUD). The experiment consisted of three stages: the first stage employed either a psychological trigger for national pride or a control. Heart rate variability monitors were employed to observe physiological changes and reactions to the framing stimulus. Since national pride is a psychological experience towards the national identity, it was important to observe any physiological changes produced when activating this emotion. It was also important to observe the variance in different physiological changes between the control group and the treatment group. The second stage was an incentivised tax compliance experiment. During the final stage, socio-demographic and associated information was gathered via a questionnaire administered after completion of the experiment, while students waited for their payment to be calculated and prepared. The participants answered questions relating to their citizenship status, feelings during the experiment, opinions about cheating on welfare, and other relevant topics (see S22–S32 Figs). The responses to the questionnaire were not forced, so some observations are missing from this section. The experiments were programmed and executed with CORAL [27], a lightweight and flexible framework for laboratory experiments developed as a user friendly, adaptable, and stable alternative to Z-tree; across nine sessions it did not crash or result in unusable data.

### 2.2. Introduction stage

Participants received instructions on how to attach the heart rate monitors with three single-use electrodes which were placed on each person’s chest. Heart rate monitors were then individually checked by the experiment administrator to ensure they were functioning correctly.

Communication between participants was forbidden during the experiment, however questions to the experiment administrator were permitted. When participants started the experiment, they were met by the screen (S5 Fig). Students were given a hand-out of the experiment instructions (S18 Fig), and instructions were also displayed on the computer screen (S6 Fig). All instructions were presented in the same language (English), and a shorter set of instructions were read out by a native English speaker. This ensured that everyone received the same instructions and it gave participants the opportunity to clarify the instructions. It also ensured that everyone knew that all participants received the same instructions and the same information. Importantly, the main experimenters were the same across all sessions, which eliminated possible variations arising from uncontrolled procedural differences or uncontrolled personal differences between the experimenters.

The participants were informed that this was an experiment in the economics of decision making, and that they could earn real money by taking part in the experiment. They were also told that their decisions would affect the amount of money earned. Before beginning the income earning stage of the experiment, the participants went through a trial round to ensure they understood the instructions, particularly the income earning and tax payment task. During this trial round (S7 Fig), the experimental taxpayers engaged in the same type of problem-solving tasks repeated in the 20 rounds (years) of the income earning task.

The next step gave participants practice in declaring their income (S8 Fig), before showing the results of their decisions (S9 Fig). After successfully completing the trial round, the experiment moved to a confirmation screen, at which point the participants were asked to check whether they understood the experiment and were happy to proceed with the next stage (S10 Fig).

### 2.3. Psychological framing

To ensure that all students were exposed to the psychological framing at the same time, the experiment was programmed to wait for all students to register their understanding of the instructions. As some students completed the trial round faster than others, a wait screen was displayed to give the others a chance to progress to the same stage (S11 Fig).

Once all students had indicated that they understood the instructions, the experimenter showed a short video to introduce the psychological framing. There were three versions of the framing video, each of equal length. The first depicted “iconic” images of Australia, including Uluru, the Opera House, the Sydney Harbour Bridge, the national flag, the Outback, and the Great Barrier Reef. This video was set to an orchestral version of the national anthem “Advance Australia Fair”. The second version of the video was based on great and memorable Australian sporting moments primarily from the previous 10 years (but also including the record-breaking 4x100 metre men’s relay at the Sydney Olympics in 2000). This video was composed of clips such as the Socceroos qualifying for the World Cup in 2006, and then again in 2013, as well as Sam Stosur winning the US Grand Slam Tennis Championship. This “sport” video was set to the same instrumental version of the national anthem as the “icon” video. The third video was a control video, with a neutral themed Adagio by Mozart (Oboe Quartet in F Major, K. 370), set to the visualisations automatically provided by iTunes; however, as pointed out by an anonymous referee, using unrelated pictures in the control framing may potentially be a better alternative to the iTunes visualisations. An example of the type of random moving patterns encountered by the students in the control can be seen in the S12 Fig, along with example screenshots of the two treatment videos. The video was produced by pre-recording the visualisations on a desktop to ensure that each screening of the control video was exactly the same. The control, “icon”, and “sport” videos were shown in four, three, and two experimental sessions, respectively.

### 2.4. Real effort task/income earning stage

Once the video had finished, the students were reminded again of the real effort task involved (S13 Fig). Participants were then presented with their first set of 10 problems to solve within the 20 second time frame, the result of which would determine their income for that round.

Students received a minimum payment of $5 for participating in the experiment. This lump sum was distributed over the 20 rounds of the experiment and resulted in a tax adjusted 36 cent endowment per round. Students earned additional income by answering a series of simple 3-digit problems. Students were given 20 seconds to answer as many as possible. Using such an endogenous allocation of money based on their abilities could provide a stronger test of their compliance, as “earning” the money reinforces the idea of personal achievement and increases the costs of handing over the money. Requiring this effort in earning income further approximated reality as the students were making tax compliance decisions based on their income rather than perhaps treating it as an endowment with which they could gamble against the possibility of an audit (see Durham et al. [28] for an overview of how different income sources affect tax compliance).

### 2.5. Income declaration task

Participants were then asked to declare their taxable income. Some participants raised questions regarding this step. In all cases the students were given the same answer: “It is tax time, and it is time to declare your income. It is your decision”. To the extent that it was possible, the experimenter did not deviate from this information.

The computer screen then displayed information advising the student of the outcome of that round, including how much tax they had paid. This same procedure repeated over the 20 rounds of the experiment, and the students were aware from the beginning of the experiment that there would be 20 rounds, or years, during which they would earn income and accumulate wealth. Of course, full defection was possible, and participants could declare no income if they wanted to avoid paying taxes completely (S14 Fig).

If the participant chose full compliance, they declared their total income for the period (S15 and S16 Figs). A variable called *tax compliance ratio* was generated that measured the ratio of the income declared by the participant to the actual income earned. This measure is calculated in each round. Tax compliance ratio becomes the main dependent variable (intensive margin) in the following econometric analysis on the effect of the treatments and HRV. Additionally, we also provide supplementary analysis based on the extensive margin (S2 Fig); i.e., by creating a dummy variable indicating whether all earned income was declared (full compliance).

Initially, an additional ‘treatment’ was included to test whether knowing the destination of the tax payments made a difference to tax compliance. The treatment alternated between two cases: in the first, students were explicitly told that taxes would be paid directly to the Australian Taxation Office (ATO). In the second, there was no mention of any destination for the taxes, and students were told only that the tax they paid on their earnings would be deducted from their accumulated wealth. After the first few sessions it became clear that it did not matter to the students whether the instructions were explicit about paying the tax or not (there was no effect on the final payments). For example, the average compliance ratio does not statistically differ between sessions with and without the ATO treatment in the control frame (t = 0.44, p = 0.661) as well as in the treatment frame (t = 1.2, p = 0.229). For recent literature on the effect of tax payments’ destination on compliance behaviour, see e.g., Bellemare et al. [29].

However, the ATO treatment was retained as it added realism to the experimental setting, and maintained social distance from the experimenter. In total, six sessions were conducted with the ATO treatment and three sessions without. To comply with the ethics requirements of the QuBE lab and QUT generally (which prohibit experimenters from deceiving participants or telling them something that is not true during the course of the experiments), the taxes were actually paid to the ATO for the sessions in which students were advised of the destination for their tax payments (a money order for a total of $469.61 was forwarded to the ATO after completion of the nine experiment sessions).

The likelihood of audit in each round was 10 percent. There was no endogenous audit selection rule; that is, every participant in each round was equally likely to be selected for an audit. If students were found to have cheated on their declared income, they were fined 1.5 times the amount of tax they owed in that round (year), which was deducted from the income earned for that round (see S17 Fig for screenshot of audit outcome).

### 2.6. Questionnaire

At the end of the 20 rounds, participants were asked to complete the questionnaire, after which they would be paid and leave the lab (see S22–S32 Figs for screenshots of questions). The students were not forced to respond to all items on the questionnaire, which led to some missing data. While this is clearly not ideal, there is a trade-off between the decision to force responses to questions and the possibility of dealing with noisy data [30].

### 2.7. Physiological measurement

Established protocol from previous QuBE experiments was followed when measuring heart rate variability (HRV) [20, 21, 31]. One advantage of using the HRV monitor to record physiological data is its portability, which overcomes one of the major disadvantages of fMRI technology–the size of the equipment. The nature of fMRI studies restricts the number of participants that can be involved in an experiment, as well as limiting the interactions between participants, where the experiment design calls for interaction [31]. In contrast, small, portable HRV devices are non-intrusive and record HRV parameters with medical levels of accuracy on a large number of participants simultaneously [31, 32]. As with previous experiments, [20, 21, 31] HRV was recorded using the Holter Medilog Digital ECG Recorder AR4. These HRV monitors have a high sampling rate of 128Hz. The devices also measure respiration rates and have an inbuilt algorithm for detection of heartbeat.

The HRV can offer some insight into the processes carried out by the vagal nerve, which itself offers bidirectional feedback between the brain and the visceral organs, impacting the autonomous nervous system (ANS). The ANS is made up of the inhibitory parasympathetic nervous system (PNS) and the excitatory sympathetic nervous system (SNS), the operations of which can be measured via variability in the heart rate (HRV) [33, 34]. The ANS has two major yet physically conflicting purposes: it monitors and takes care of the internal organs, and responds to “external challenges” [35, p 80]. The SNS is dominant during physical or psychological stress [36], and “affects the heart rate indirectly through the sympathetic nerves” [31, p 119]. In contrast, the PNS is generally dominant during resting, relaxing, digesting, and social engagement behaviours, promoting growth and restoration [35, p 80]. The PNS affects the heart directly through the vagal nerve. Porges [36, p 302] suggests that HRV “may provide a portal to the dynamic assessment of vagal function”, and further, that “beat-to-beat heart rate variability is a window to the neural regulation of the heart”. However, Porges notes that the ability to reach any conclusions regarding HRV and its emotional correlates relies on the ability to accurately measure, quantify, and interpret HRV, as well as knowledge of the underlying neural mechanisms [37].

This study is specifically interested in measurements of the frequency of HRV (explained in Appelhans and Luecken [33]). In controlled environments, individual stress levels (LF/HF ratio) can be identified using ANS’s control over HRV [31, p 122]. The SNS is related to the LF (low frequency, [0.033–0.15 Hz]) and PNS is related to the HF (high frequency, [0.15–0.4 Hz]), and to respiratory sinus arrythmia [33, p 233]. As explained by Dulleck et al. [31] and Appelhans and Luecken [33], the ratio of LF to HF activity may serve as an indicator of psychological state; representing the relative dominance of SNS to PNS in the control of the heart. We anticipate that with measurement of HRV, we gain an insight into the activities of the nervous system and may even be able to pinpoint the physiological correlates of emotions.

A standard time-frequency analysis approach is followed to obtain the LF/HF ratio from the interpolated heart-beat data [31, 33]. We report results using the Autoregressive method (AR), but the results remain stable over different methods (FFT, AR and Wavelet transform).

### 2.8. Analyses

We describe the effect of the psychological framing treatments (exposed to *icon* and *sports* video) on participants’ decisions in the tax experiment, namely, the *tax compliance ratio*. When comparing the tax compliance ratio across groups, we perform mean comparison test with *p*-values adjusting for multiple comparison using the Tukey’s method, which conservatively accounts for the elevated type I family-wise error rate. Since the outcome variable of interest is bounded between 0 (non-compliance) and 1 (full-compliance), we also perform the Wilcoxon–Mann–Whitney rank sum test with multiple comparisons adjustment following the Dunn’s test procedure. While we report only the results from the mean comparison tests, the result from the nonparametric tests yields very similar qualitative conclusions. Treatment effects on the extensive margin of tax compliance are shown in S2 Fig.

## 3. Results

### 3.1. Effect of trigger and nationality on tax honesty

Before exploring nationality differences, we checked the general effect of the different treatments on the average tax compliance ratio (see S1 Fig). Overall, there is a positive effect of the *icon* treatment on tax honesty in the pooled sample. That is, participants in the treatment using iconic images to trigger national pride demonstrated a higher level of compliance (Mean = 0.74; 95% CI: 0.71–0.77) relative to those in the control, with a 9.6 percentage points difference (p < 0.001). One potential to consider here is whether participants may associate the objects in the “iconic” images with how tax money was spent (e.g., the Opera House and the Harbour Bridge and the preservation of natural monuments such as the Great Barrier Reef or Uluru), which may lead to change in tax compliance. For example, knowledge and transparency on public expenditures are associated with tax compliance [38]. However, as the framing happened before the tax setting, it may be less likely that such a connection was formed.

Surprisingly, those in the *sport* treatment (M = 0.60; 95% CI: 0.57–0.64) reported a relatively lower level (4 percentage points) of tax compliance than those in the control, however this effect was not statistically significant (p = 0.14). The average tax compliance ratio for the control treatment was 64.3% (95% CI: 61.8–66.7%).

**Fig 1:**
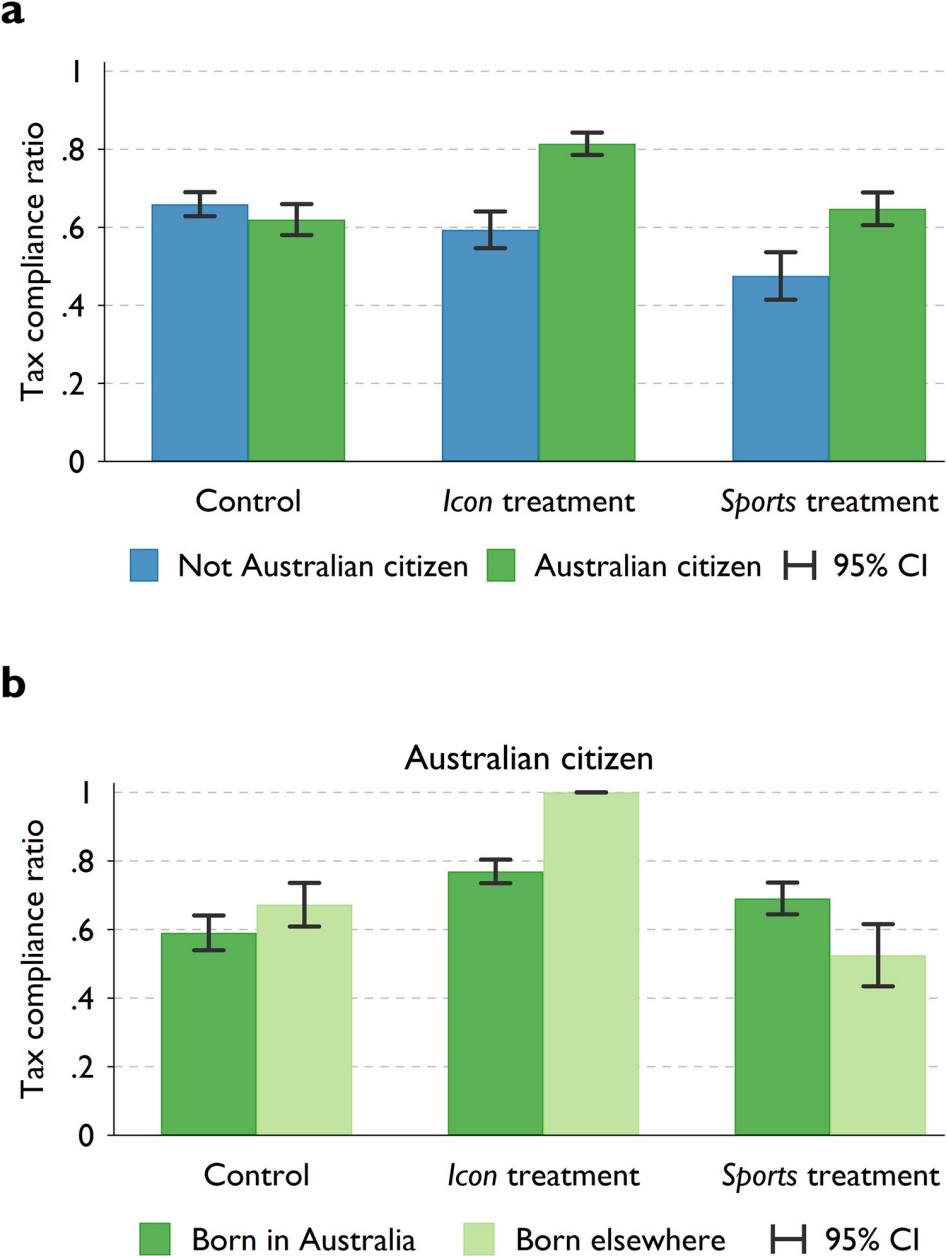
Tax compliance by treatment and citizenship status. Panel **a** shows the average *tax compliance ratio* for participants in the control, *icon* and *sport* treatment group, decomposed by participants’ citizenship status. Number of participants: *N*_*control*,*non-Aus*_ = 35; *N*_*control*,*Aus*_ = 25; *N*_*icon*,*non-Aus*_ = 16; *N*_*icon*,*Aus*_ = 31; *N*_*sports*,*non-Aus*_ = 8; *N*_*sports*,*Aus*_ = 23. Panel **b** shows the average *tax compliance ratio* of *Australians* participants decomposed by country of birth, i.e., born in Australia and born elsewhere. Number of participants: *N*_*control*,*Aus born*_ = 16; *N*_*control*,*born elsewhere*_ = 9; *N*_*icon*,*Aus born*_ = 25; *N*_*icon*, *born elsewhere*_ = 6; *N*_*sports*,*Aus born*_ = 17; *N*_*sports*,*born elsewhere*_ = 8. Error bars represent 95% confidence intervals.

To determine whether Australian citizens display a stronger reaction to the treatments than non-Australians, in Fig 1A we report and compare the means of tax compliance ratio, distinguishing the sample between treatments and citizenship status. In general, we find the treatment effects on the fraction of full tax compliance (extensive margins) to be highly comparable to the main results (see S2A and S2B Fig). This suggests that the treatment differences are not due to amount of income evaded by the subjects but rather driven by the number of subjects evading income.

We observe that Australian citizens in both the icon and sport treatments designed to trigger national pride reported higher levels of tax compliance than non-citizens in the respective treatment groups. Specifically, Australians reported 22.1 (95% CI: 0.14–0.30; p < 0.001) and 17.2 (95% CI: 0.06–0.28; p < 0.001) percentage points higher tax honesty than non-Australians in the icon and sport treatment, respectively. When we pooled the icon and sport treatments, treated Australians reported 18.9 pp higher tax compliance ratio than treated non-Australians (95% CI: 0.13–0.25; p < 0.001, S2C Fig).

This substantial contrast is highlighted by the statistically insignificant difference in the level of *tax compliance* between *Australians* and *non-Australians* in the control group (difference of 3.9 pp, 95% CI: -0.11–0.03; *p* = 0.59). We also find that the *tax compliance ratio* of *Australians* in the *icon* treatment is, on average, 19.4 (95% CI: 0.12–0.27; *p* < 0.001) percentage points higher compared to *Australians* in the control group, whilst *non-Australians* exposed to the *icon* treatment show lower levels of *tax compliance* to their counterpart in the control group, although the difference is not statistically significant (difference of -6.57 pp; 95% CI: -0.15–0.015; *p* = 0.19). Although we did not find a significant effect on *Australians* who were exposed to the *sports* video (average difference in compliance ratio compared to *Australians* in the control group is 2.76 pp; 95% CI: -0.05–0.105; *p* = 0.911), *non*-*Australians* reported a considerably lower level of compliance in the *sport* treatment compared to their peers in the control group (difference of -18.4 pp; 95%CI: -0.29– -0.08; *p* < 0.001). *Non-Australians* may experience the *sport* treatment as an adversarial situation due to its competitive nature and finding themselves in the outgroup, this, in turn, may lower their willingness to cooperate with the group to which they do not belong.

In Fig 1B we distinguish between *Australian* participants who were born in Australia (*n* = 58) and those born elsewhere (*n* = 21). We find that the *icon* treatment has a similar effect on Australians who were born in the country as they report a higher level of *tax compliance* than those in the control group (difference of 17.9 pp; *p* < 0.001). Interestingly, locally born *Australians* also demonstrate a higher level of tax honesty in the *sport* treatment compared to their counterparts in the control group (difference of 10 pp; *p =* 0.024). Furthermore, while *Australians* in the control group reported similar levels of tax compliance regardless of their country of birth (*p* = 0.276), those who were born outside of Australia reported higher level of tax compliance in the *icon* treatment (difference of 32.8 pp; *p* < 0.001) but lower level in the *sport* treatment (difference of -14.7 pp; *p* = 0.032) compared to the those in the baseline. However, due to the small number of observations among Australian participants born elsewhere, one should exercise caution when interpreting these results.

**Fig 2:**
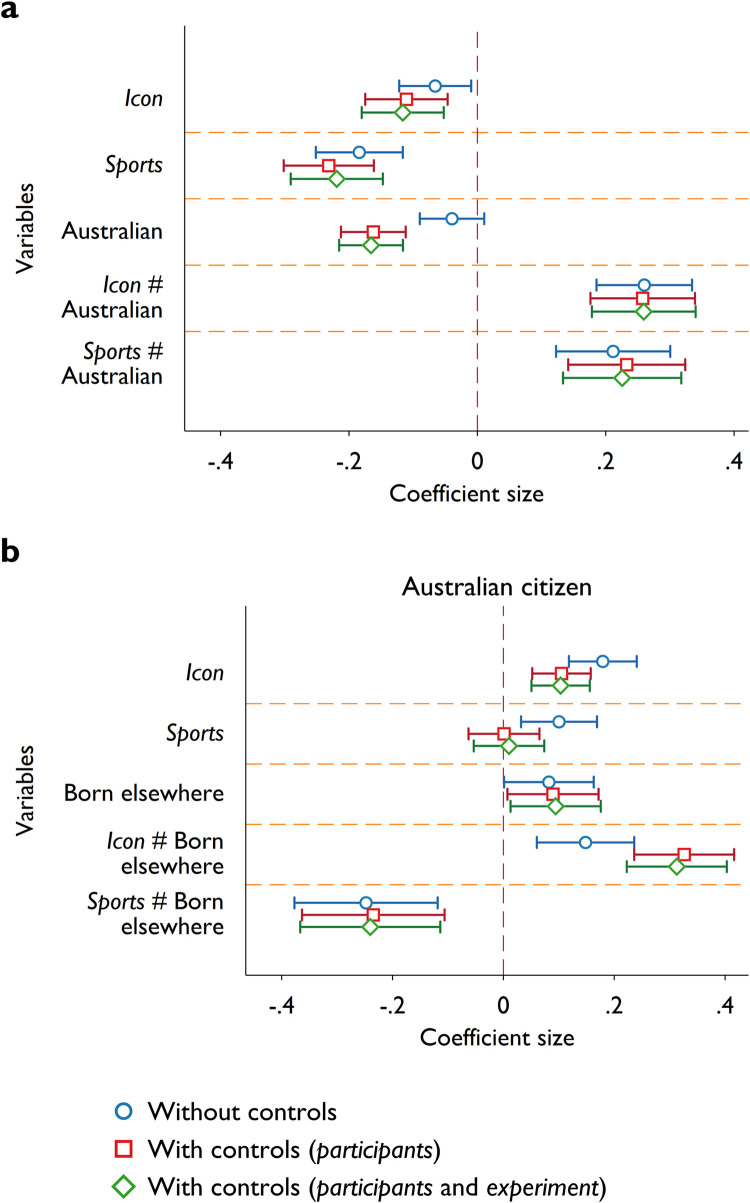
Effect of video framing on tax compliance. Panel **a** shows the ordinary least squares (OLS) regression coefficients of the interaction terms between the treatment variable (*icon* and *sport*) and citizenship status (*Australian*) on participants’ *tax compliance ratio*. In panel **b**, using the *Australian* sample, we show the coefficients of the interaction terms between the treatment variable and a dummy variable which indicates if the participant was not born in Australia. Blue circles represent the point estimates of the unconditional regressions without controlling for other factors. Red squares show the point estimates from regressions that control for participant’s age, gender (*female* dummy), church attendance, field of study (*Business* dummy), degree (*post graduate* dummy), and disposable income. We further control for experimental features such as round fixed effects, and whether the participant was audited or fined in the last round (green diamonds). Error bars represent 95% confidence intervals based on robust standard errors.

We also ran a set of ordinary least squares regressions to assess the robustness of our findings, taking into consideration effects from individual heterogeneity, namely, participants’ age, gender, church attendance (dummy variable), field of study (*Business* dummy), degree (*post graduate* dummy), and disposable income. We further control for features in the tax experiment, i.e., round fixed effects, whether the participant was audited or fined in the previous round. We present the coefficient estimates of the treatment effects and the interaction terms between the treatment variable (*icon* and *sport*) and citizenship status of the full sample (Fig 2A) and country of birth (born in Australia) with the *Australian* sample (Fig 2B). It is evident that the coefficients of interest are quite stable to the addition of control variables and the effect size is comparable to the simple mean comparison tests.

### 3.2. Interaction of HRV with trigger and nationality

**Fig 3:**
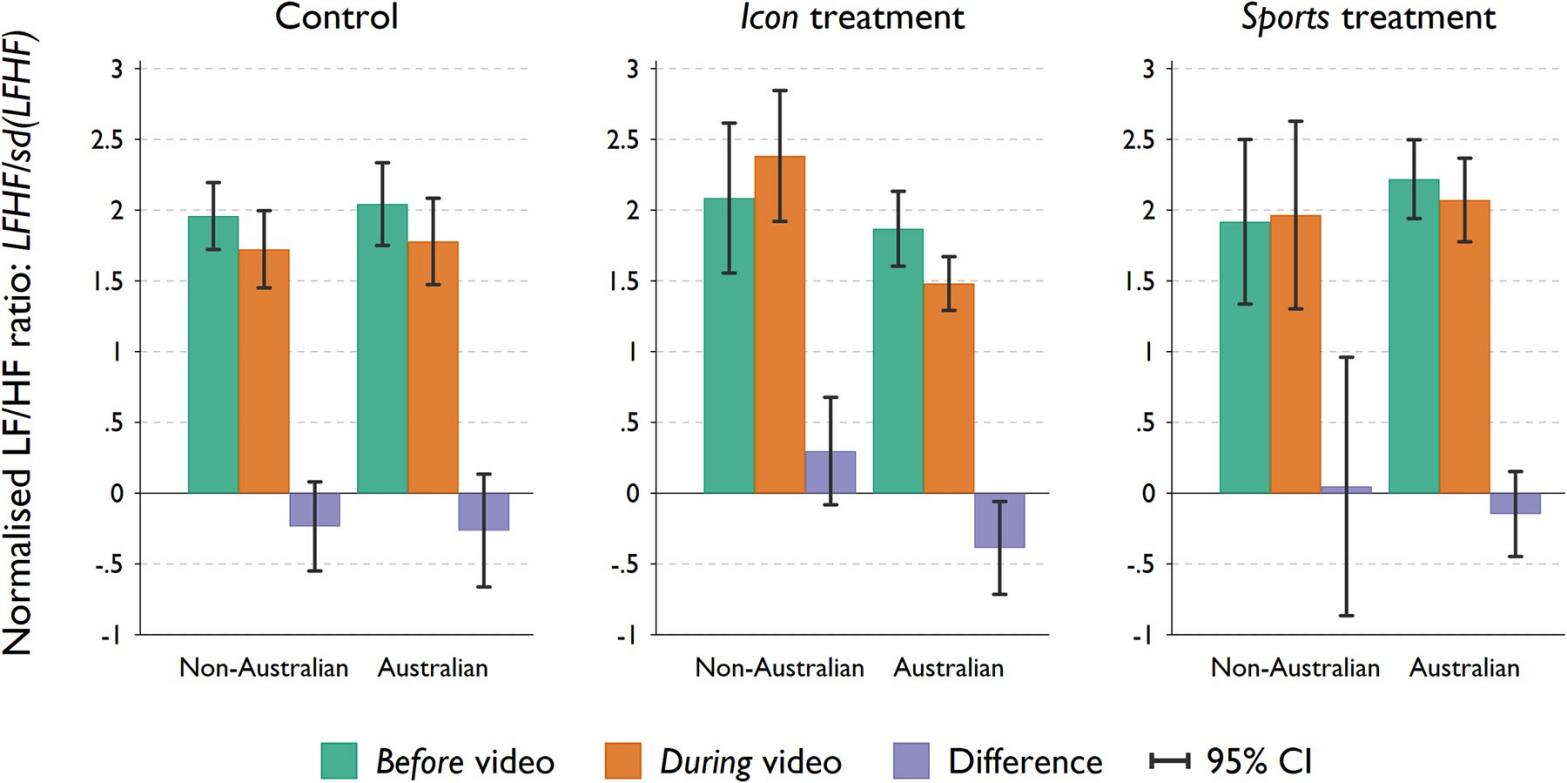
HRV and change in HRV before and during the video triggers (by citizenship status). Comparison of the average normalised LF/HF ratio (stress indicator) before and during the video framing to determine how participants react to the trigger/treatment. The green bars indicate the average LF/HF ratio before the video, the orange bars indicate the LF/HF ratio during the video, and the purple bars indicate the difference. A negative difference in the purple bars indicates that the LF/HF ratio decreased, due to a relative change in the PNS/SNS ratio, which indicates that participants were relaxed by the treatment. Error bars represent 95% confidence intervals.

The results presented thus far have yet to consider participants’ physiological responses; namely, how participants’ heart rate variability changes while watching the framing video. Hence, in Fig 3 we compare the normalised LF/HF ratio (stress indicator) *before* and *during* the video to determine how participants react to the trigger/treatment. The LF/HF ratios were calculated by averaging the 1-second interval LF/HF readings across a period of 160 seconds during the video and 20 seconds before the start of the framing video, respectively. These ratios (and their difference) are normalised by the standard deviation of the 1-second interval LF/HF ratio in the before video period. The distribution of (SD) normalised 1-second interval LF/HF ratio is slightly right skewed. Hence summarising the LF/HF ratios (and the differences) using the average might be sensitive to spikes in the data. We thus report the median normalised LF/HF ratios before and during the video and the corresponding change (S3 Fig in the Appendix); however, the results are very similar to that of Fig 3.

The green bars indicate the average normalised LF/HF ratio before the video, the orange bars indicate the average normalised LF/HF ratio during the video, and the purple bars indicate the difference of the two. A negative difference in the purple bars indicates that the LF/HF ratio decreased during the video, due to a relative change in the PNS/SNS ratio, which indicates that participants were relaxed by the treatment.

The control treatment relaxed both *Australians* and *Non-Australians* similarly (two-tailed *t*-test on the change in LF/HF ratio between the two samples: *p* = 0.906). On average, participants in the control treatment experienced a decrease in the normalised LF/HF ratio in response to the video (two-tailed *t*-test with pooled sample; *p* = 0.043). It suggests an increase in the HF activity and activation of the parasympathetic nervous system (PNS). Such a result is not surprising and is supported by other studies on the relaxing effect of music in general [39]. We also perform the binomial probability test to assess whether the proportions of participants with increased or decreased HRV are different to 0.5 (which would suggest equal number of participants were excited or relaxed by the treatment video). The results are qualitatively the same as the one-sample *t*-test. Nevertheless, when the two samples were tested individually, neither is statistically significant (*p*-value equals to 0.183 and 0.137 for *Australians* and *non-Australians* in the control group, respectively).

In the treatment designed to trigger national pride using iconic images (*icon*), only the *Australians* experienced a statistically significant decrease in the LF/HF ratio (*p* = 0.023). Statistical significance is obtained for a *one*-sided binomial probability test (*p* = 0.06) on the proportion of *Australians* with decreased LF/HF ratio (66%).

The *non-Australians* actually experienced the opposite reaction; although, this difference is not statistically significant (*p* = 0.114). Nevertheless, the difference in change of LF/HF ratio between the two samples is statistically significant (two samples two-tailed *t*-test: *p* = 0.012). Interestingly, the *Australians* exposed to the national pride trigger of *sports* experienced (on average) a slight decrease in the LF/HF ratio, indicating that they were slightly relaxed by the video; however, the difference is not statistically significant (*p* = 0.317). Lastly, we do not find any statistically significant difference in LF/HF ratio for *non-Australians* in the *sport* treatment (*p* = 0.906), nor is the difference statistically significant from other *Australians* in the same treatment (*p* = 0.56).

Furthermore, using self-reported levels of national pride, we examine whether the physiological responses from the treatment triggers are more pronounced for *Australians* who have a higher level of national pride. To this end, we examine the coefficients of the interaction terms between treatment groups and self-reported national pride (5-point Likert-scale) in a regression on the changes in the LF/HF ratio, using only the *Australian* sample (*n* = 68). We find that the relationship between the average changes in the LF/HF ratio–relative to the *control* group–is more positively associated with self-reported national pride in both *icon* (*b* = 0.317, *p* = 0.063) and *sport* (*b* = 0.622, *p* = 0.009) treatment groups. This suggests that *Australians* with higher national pride are less relaxed/more excited when exposed to the triggers and compared to those in the control group.

An additional test that could have been considered is whether participants are receptive to the physiological framing by testing whether the feeling of national pride has increased after exposing to the video stimuli. However, this approach is not feasible as national pride was not measured prior to the framing. Moreover, asking participants to self-report their level of national pride before and after the priming may suffer from measurement issues such as internal consistency. Also, because national pride was assessed after the tax experiment, the framing effect may have worn off due to the time passed. In general, how long the psychological framing effect lasts is an interesting question to be explored by future research. Nevertheless, for this study, we observe that *Australians* who were exposed to the *icon* or *sport* treatments report, on average, higher national pride. However, these differences (compared to those in the control group) were not statistically significant (*icon*: *b =* 0.232, *p* = 0.459 and *sport*: *b =* 0.113, *p* = 0.669, respectively). On the other hand, we also find that *non-Australians* in the *sport* treatment report lower values to the statement “*I feel closely connected to Australian people*” but the difference is not statistically significant (*b* = -0.379, *p* = 0.2).

### 3.3. HRV responses to trigger, nationality, and tax honesty

To separate participants into those that showed a strong physiological reaction and those that did not, treatment specific cut-offs were introduced. The (within group) analysis of the changes in the LF/HF ratio (Section 3.2) made it clear that a single cut-off could not be chosen globally due the large heterogeneity of subjects’ reactions to the different treatments. Hence a suitable cut-off was selected based on a visual inspection of the relationship between the HRV data and the tax declaration behaviour of the participants in the experiment with the intent to maximise the differences between affected and not-affected participant groups. In other words, the cut-offs were chosen to maximise the difference in the result of interest (between those who are above the cut-off to those who are below) for each treatment group (data-driven approach). However, this also means that statistical inference (e.g., p-values) is no longer meaningful.

For the control session, the cut-off for changes in the LF/HF ratio is set to -0.4 since in general participants experienced a relaxing effect (30 subjects with above the ΔLF/HF cut-off). For the *icon* and *sport* treatment, cut-offs were chosen to be 0.6 and 0.5, respectively (8 subjects with above the ΔLF/HF cut-off in both treatments).

**Fig 4:**
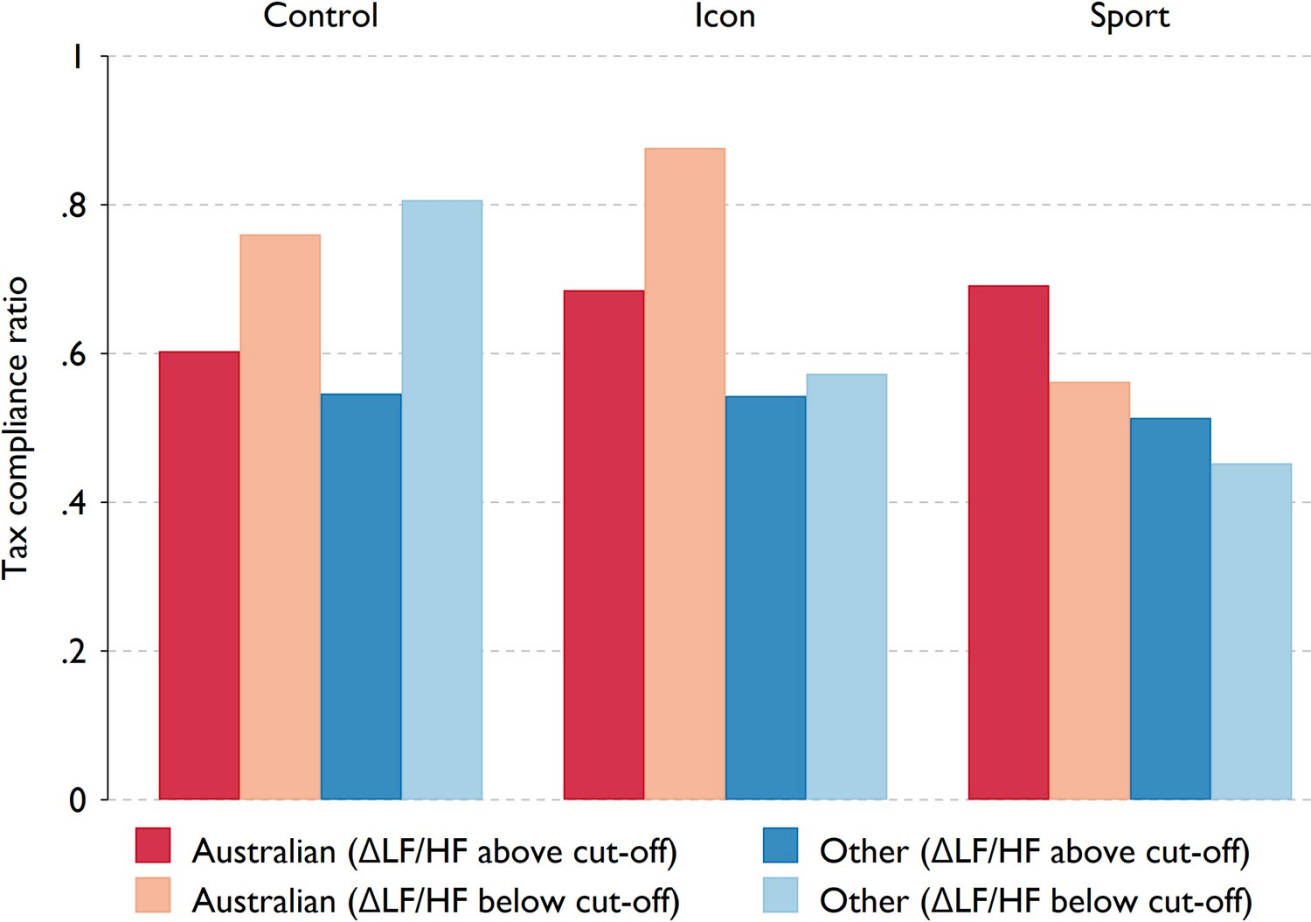
Tax compliance by treatment, citizenship status, and HRV. The red bars indicate the tax compliance ratios of Australians who experienced a relative increase (darker shades) in the LF/HF ratio above the treatment specific cut-off and who demonstrated a relative decrease (lighter shades) in the LF/HF ratio (below the cut-off), respectively. The blue bars are tax compliance ratios of the non-Australians who were excited (darker shades, ΔLF/HF above treatment specific cut-off) and relaxed (lighter shades, ΔLF/HF below cut-off) by the video.

In Fig 4, we show the difference in the levels of tax compliance according to whether the participants are *Australian* or *non-Australian*, and also whether they were excited or relaxed by exposure to the treatment; i.e., exhibiting changes in the LF/HF ratio that were above or below the treatment specific cut-offs. It is clear that both *Australians* and *non-Australians* whose change in the LF/HF ratio were below the control treatment cut-off (relaxed) demonstrated higher *tax compliance* (left panel). Perhaps it is not surprising that some participants were relaxed by a neutral video set to a piece by Mozart, although there are mixed results from studies that attempt to prove or disprove the so-called “Mozart effect” (see Burns et al. [40] and Chabris [41] for a discussion on the differing perspectives). Nevertheless, it is intriguing that our participants who were relaxed by the control video exhibited a higher tax compliance than the participants who were not relaxed by the same video.

We also see that Australians for whom the ΔLF/HF ratio fell below the *icon* treatment cut-off (relaxed) demonstrated a higher level of tax compliance (light red bar) with a difference of 19.1 percentage points, compared to Australians who exhibited a relative increase in LF/HF ratio in the same group, as well as Australians in the control group (difference of 27.3 pp, and 11.6 pp compared to those with increased and decreased LF/HF ratio, respectively).

This effect is important, because while participants who were relaxed in the control group showed higher tax compliance irrespective of citizenship, in the *icon* treatment only Australians that were relaxed showed higher tax compliance. On the other hand, the Australians with ΔLF/HF ratio above the *sport* treatment cut-off (indicating–in relative terms–an activated SNS) also demonstrated high levels of tax compliance (mean = 0.69). This group is denoted by the darker red bar in the right panel of Fig 4, indicating Australians whose LF/HF ratio increased above the cut-off in response to the *sport* video. This effect is also important as it shows a large contrast to the patterns observed among participants in the control group. Additionally, Australians with ΔLF/HF ratio below the cut-off in the *sport* treatment showed lower tax honesty (lighter red bar) compared to their peers who were relaxed in the control group (difference of 19.8 pp). Such results might be understandable from an ingroup/outgroup or even an evolutionary or biological perspective. Australians have been socialised through exposure to these iconic landscape images, and when presented with this video set to the national anthem, are able to recognise the images and music as belonging to their ingroup. They are part of the group this video represents, and thus their neuroception in orienting to this environment would assess that the environment is familiar, and safe (see discussion regarding Porges [35]). Activating their PNS and allowing relaxation–and promoting social engagement behaviours–could account for the increased tax honesty or tax compliance. Non-Australians are not part of this ingroup and may not feel the same sense of familiarity or socialised prompt to contribute to the group. Furthermore, the *sport* treatment activated the SNS in Australians, with the neuroception assessing a challenging situation (competition with the outgroup), although Australia was winning in all scenarios presented (enhancement of cooperation through ingroup feeling). The physiological response was still one of reaction to a challenge, with increased SNS activity. Australians who were excited by the video exhibited higher tax compliance/tax honesty, suggesting there are at least two different channels through which national pride and national identity may be activated to invoke the ingroup belonging and encourage increased tax compliance. That is, for members of the ingroup, increased tax compliance may be correlated with increased PNS activity, and increased tax compliance may also be correlated with increased SNS activity. The results of this study have found not only one possible physiological marker of national pride, but potentially have identified two such markers.

### 3.4. Socialisation

**Fig 5:**
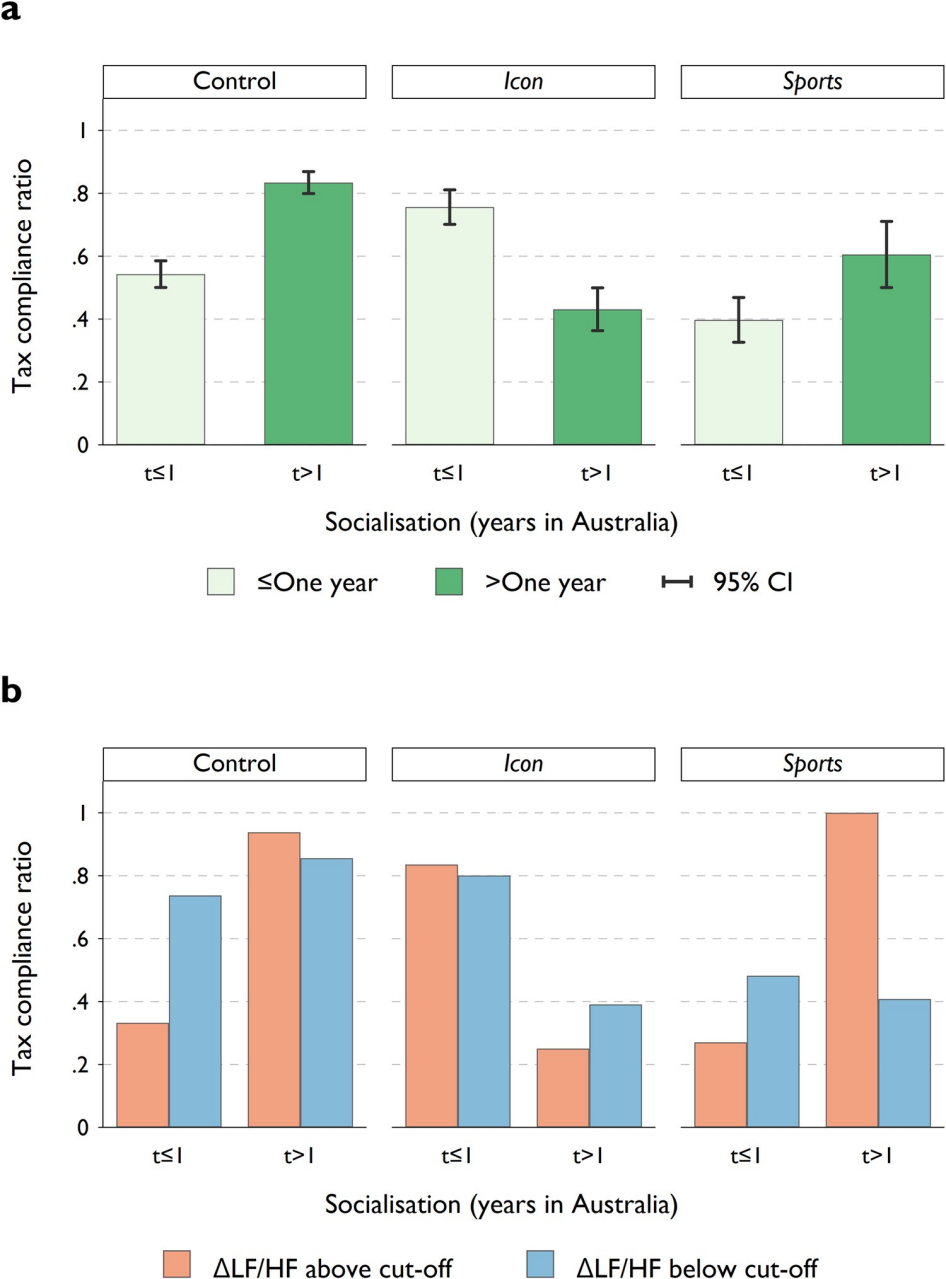
Tax compliance by treatment, duration of stay in Australia, and HRV. Panel **a** shows the average *tax compliance ratio* of *non-Australian* participants by their length of stay (over one year or less) in each treatment group. Error bars represent 95% confidence intervals. In panel **b**, we further separate the sample into two groups by their physiological reaction to the framing video (ΔLF/HF ratio above or below cut-offs).

Next, we explore the effect of the treatments on participants in the *non-Australians* group. The response might be mediated by socialisation, as measured by the duration of time in Australia (although, the level of socialisation and integration may naturally differ from person to person). We thus categorise *non-Australians* participants into two groups based on their length of stay in Australia, namely, *less than or equal to one year* (*n* = 34) and *more than one year* (*n* = 25). In Fig 5A, we show the effect of the framing videos on *tax compliance* of *non-Australian* participants, grouped by the length of socialisation. Fig 5B further breaks the samples into those with changes in the LF/HF ratio above or below the treatment specific cut-offs while exposed to the framing video. Again, here we note that due to the small sample size, one should exercise caution when interpreting results, whether estimates are statistically significant or insignificant.

In general, *non-Australians* who stayed for more than a year (control group) report a higher level of tax compliance (regardless of whether they were excited or relaxed). This could be linked to a higher level of social integration. The *sport* treatment does not seem to lead to a strong crowding out effect for those who have been in Australia for more than 1 year. It is less clear why a prolonged stay in Australia would negatively affect compliance in the *icon* setting. When looking at the HRV, we observe some similarities between Australians and those born overseas who have been in Australia for a longer period (S4 Fig). Excitement in the *sport* setting promotes *tax compliance*. However, contrary to *Australians*, a relaxing effect of the *icon* treatment is not correlated with higher levels of honesty.

## 4. Discussion

Tax compliance is an interesting topic as it fundamentally affects the ability of a government to collect the revenue necessary to provide the services and infrastructure we require in a “civilized society”. From this perspective, the willingness to pay taxes is of crucial importance to policymakers, and identification of factors that increase compliance is the topic of a thriving research agenda. Based on the analysis conducted in this paper, it seems possible that certain methods of socialisation might engender a feeling of national pride (identified by measurable physiological processes), thereby increasing the level of tax compliance in some citizen taxpayers. However, not all citizens react in the same way to the same treatment, so national pride could be important for taxation only if people subscribe to the concept. Using psychological framing and measuring heart rate variability, we found evidence of a link between activated national pride and tax compliance, which is a valuable follow up from the survey results available in previous literature. One of the innovations of this paper is its use of heart rate variability (HRV) measurement in a standard tax compliance experiment to explore national pride and as a way of obtaining an external visualisation of national pride.

The results of this study indicate that both Australians and non-Australians who were relaxed by the control video (Mozart and iTunes visualisations) exhibited higher tax compliance. Australians demonstrated higher tax compliance when they were relaxed by national icon images and the national anthem (low LF/HF ratio), experiencing a PNS dominant reaction to the psychological framing. Familiar faces or voices can encourage a neuroception or orienting evaluation of a safe environment. It is also possible that familiar music and images could promote this neuroception of safety, due to priming from the national anthem included in the experiment videos. Australians also reported higher levels of tax compliance when they were excited by images of sporting victories set to the national anthem (high LF/HF ratio), which suggests two different pathways for the expression or activation of national pride. It also suggests that the results are not due only to the propensity of more easily relaxed individuals to be more compliant.

Initially, we were looking for a physiological marker of the emotion of national pride, but we now have evidence of two channels through which national pride affects the individual and their emotions. That is, there are two different sets of physiological measurements or patterns of responses that could be correlated with the psychological phenomenon of national pride. Obtaining these types of measurements may eventually serve the same purpose as the established correlation between increased heart rate, release of cortisol, and anxiety. In future, if a person experiences certain externally measured physiological responses, there might be a clearer idea regarding the internal processes. In doing so, the link between emotions and actions (or possibly even motive and behaviour) can be glimpsed, by observing the neurophysiological correlates of attitudes or emotions using data obtained from devices such as heart rate monitors. As Porges [35, p 76] notes: “…whether we are talking about feelings, emotions, states, or moods, we are always attempting to describe the internal states that are continuously being monitored and regulated by the nervous system”. As these internal states change, as they are monitored and regulated by the nervous system, they produce physiological markers that can be measured externally.

This paper has suggested that identifying with an ingroup at the national level is important for tax compliance, an observation that is in line with the assessment that “[t]axpayers’ identities affect their commitments and solidarities, their endorsement of certain values and goals, their internalization of social norms, and their emotions and motivations” [18, p 31]. The HRV data is analysed with respect to the observed tax compliance behaviour, which is an important innovation, as it permits investigation of the links between identity, emotions, attitudes, morale, and tax compliance. It may be that these links and factors ask for different policy responses than do the rational utility maximising or economics of crime frameworks. On this topic, Wenzel [18, p 45] draws direct implications for the tax authorities: “… social identity, specifically a sense of inclusion, can provide considerable leverage for compliance… regulators should be aware that whatever regulatory actions they take these could have negative side effects on people’s sense of identity and thus undermine compliance levels. Taxpayers who are found to define themselves primarily in inclusive terms… are more likely to feel commitment to the tax system (see Braithwaite [42]). Regulators should nurture and harness this commitment through cooperative and educational approaches”.

However, increasing tax compliance is not as simple as increasing taxpayer exposure to images or psychological priming devices such as the video treatments employed in this study. The collective social memory of the downside of excessive nationalism may have faded in the past few decades, and recent political movements indicate the extent to which we have forgotten the risks. Although the dangers inherent in government sponsored nationalistic displays were not taken seriously, Wenzel [18, p 46] was optimistic that cynical manipulation of the inclusive identity would quickly be seen as an “insincere means of control, and this, as with other forms of coercion, will likely be attributed to an outgroup” (Taylor and McGarty [43] cited in Wenzel [18]).

There are several promising directions for future research in this area. Firstly, further repetitions of the experiment would increase the sample size and allow for a robust clustering over the individual. Particularly, it will also increase the robustness of the results derived based on the subgroup analysis (e.g., participants’ nationality). Stratified sampling based on participants’ characteristics can be employed in the participant recruitment process/experimental design. Secondly, it would also be interesting to adapt the experiment for testing in different countries as each nation has a slightly different way of expressing national pride (keeping in mind that “consequences of flag exposure are not unidirectional in all countries but rather depend on the social context and the concepts people associate with the flag and other national symbols” [44, p 5].

We also found that non-Australians residing longer in Australia report a higher level of tax compliance (regardless of whether they were excited or relaxed). In addition, when looking at the HRV, we observe some similarities between Australians and those born overseas who have been in Australia for a longer period. Further research would generate more observations, allowing more robust insights into the relevance of the socialisation process by analysing different groups who have been in Australia for varying lengths of time. Moreover, it would also be interesting to follow up on those insights with a longitudinal approach–tracking individuals before and after they become Australian citizens to determine whether there is any measurable difference in their tax compliance behaviour or in-group identification. This approach could also identify whether age is an important mediating factor on the effect of group identification and socialisation. Nevertheless, future study could obtain information on non-Australian participant’s desire to live or work in Australia upon graduation as those who are more determined on settling down in Australia may be more committed to Australian national values. Thus, asking participants about their expected future length of stay in Australia or intention to migrate to Australia could disentangle such commitment effect from the socialization effect. In addition, a more detailed analysis could contribute to an understanding of how individuals acquire the rules and shortcuts for use in certain contexts (e.g., be a good citizen in the national pride context).

Other future directions for research could include the use of a more representative sample of the taxpaying public, as opposed to the standard student subject pool, as a cross-check on the validity of the results [25]. Extensions to this study could focus on self-employed individuals and those active within service industries that are characterised by the opportunity to engage in cash transactions (e.g., [15, 45, 46]). Furthermore, analysing the difference between people who construct their identity at the state, regional, local, or family level in a way that is stronger than the national identity would lead to valuable insights, as would an experiment in an area with a strong separatist movement. In addition, it would be interesting to test whether the relationship holds over time, or whether the effect of the national pride psychological framing is immediate (this would require participants to wear the HRV monitors for longer periods). The investigations listed above would also be valuable in different countries with strong regional identities and independence movements, for example, in Canada, Scotland, Spain, etc. Previous research has already provided evidence from Spain, Switzerland, and Belgium indicating that cultural and regional differences within a country affect tax morale [47]. In sum, this is likely to be a rich field of research endeavour for some time.

## Supporting information

S1 Fig Tax honesty by treatment. Effect of treatment on average *tax compliance ratio*. Participants in the treatment using iconic images to trigger national pride demonstrated a higher level of compliance relative to those in the control. Error bars represent 95% confidence intervals.
(TIF)

S2 Fig Panels **a** and **b** show the average *fraction of full tax compliance* (extensive margins of tax compliance) by treatment group (control, *icon*, and *sport*) and participants’ citizenship status. *Icon* and *sport* treatments are pooled in panels **c** and **d**, which show average *tax compliance ratio* for participants in the respective group. Panels **b** and **d** restricts the sample to *Australians* participants. Error bars represent 95% confidence intervals.
(TIF)

S3 Fig Sensitivity of HRV and change in HRV using median. The figure shows the *median* normalised LF/HF ratio (stress indicator) before and during the video framing and the corresponding difference. Error bars represent 95% confidence intervals.
(TIF)

S4 Fig Tax honesty of Australian participants by treatment, country of Birth, and HRV. The figure shows the average level of *tax compliance* of Australian participants by their country of birth (red bars represent participants were born in Australia and grey bars represent participants who were born elsewhere). Caution should be exercised when interpreting the results due to the small number of foreign-born Australian participants.
(TIF)

S5 Fig Introduction. *Screenshot of introduction screen*.
(TIF)

S6 Fig Instructions. Screenshot of instruction screen.
(TIF)

S7 Fig Earn income trial. Screenshot of earn income trial screen.
(TIF)

S8 Fig Declaration. Screenshot of trial declaration screen.
(TIF)

S9 Fig Outcome of trial. Screenshot of outcome screen.
(TIF)

S10 Fig Understand instructions. Screenshot of understand instructions screen.
(TIF)

S11 Fig Waiting for video to start. Screenshot of waiting screen before video started.
(TIF)

S12 Fig Screenshot of the treatment and control videos. Top panel (*control* video)–screenshot of visualisation generated by iTunes. Middle and bottom panels show a screenshot from the *icon* and *sport* treatment video, respectively.
(TIF)

S13 Fig Post-video information. Screenshot of post-video information screen.
(TIF)

S14 Fig Outcome of full defection in year 1. Screenshot of full defection screen.
(TIF)

S15 Fig Declaration in year 2. Screenshot of declaration screen.
(TIF)

S16 Fig Outcome of full compliance in year 2. Screenshot of full compliance screen.
(TIF)

S17 Fig Audit. Screenshot of audit outcome.
(TIF)

S18 Fig Instructions. ATO version.
(TIF)

S19 Fig Participant information. (TIF)

S20 Fig Attach HRV monitor. Instructions on attaching HRV monitor.
(TIF)

S21 Fig Attach HRV monitor. Instructions on attaching HRV monitor.
(TIF)

S22 Fig Questionnaire first screen. (TIF)

S23 Fig Questionnaire for those born in Australia. First screen of questionnaire for those born in Australia.
(TIF)

S24 Fig Questionnaire for those born in Australia continued. Second screen of questionnaire for those born in Australia.
(TIF)

S25 Fig Questionnaire for those born overseas. First screen of questionnaire for those born overseas.
(TIF)

S26 Fig Questionnaire for those born overseas continued. Second screen of questionnaire for those born overseas.
(TIF)

S27 Fig Questionnaire for those born overseas continued. Third screen of questionnaire for those born overseas.
(TIF)

S28 Fig Questionnaire for all continued. Second screen of questionnaire for all.
(TIF)

S29 Fig Questionnaire for all continued. Third screen of questionnaire for all.
(TIF)

S30 Fig Questionnaire for all continued. Fourth screen of questionnaire for all.
(TIF)

S31 Fig Questionnaire for all continued. Fifth screen of questionnaire for all.
(TIF)

S32 Fig Questionnaire for all final screens. *Final screen of questionnaire for all*.
(TIF)

## References

[1] Boulding K.E., (1992). *Towards a New Economics*: *Critical Essays on Ecology*, *Distribution*, *and Other Themes*. Economists of the Twentieth Century, Edward Elgar.

[2] Smith T. W., Jarkko L., (1998. National pride: A cross-national analysis, Chicago, IL: National Opinion Research Center, University of Chicago.

[3] Girling R., (2010). *Greed*, Random House.

[4] Konrad K.A., Qari S., (2012). The last refuge of a scoundrel? Patriotism and tax compliance. *Economica*, 79(315), 516–533. doi: 10.1111/j.1468-0335.2011.00900.x

[5] Donnelly K., (2015). Singing the national anthem at school should be compulsory. *The Sydney Morning Herald*. 27 October. https://www.smh.com.au/opinion/tolerance-of-diversity-cannot-exist-without-common-values-20151027-gkja8z.html.

[6] Butz D.A., (2009). National symbols as agents of psychological and social change. *Political Psychology*, 30(5), 779–804. doi: 10.1111/j.1467-9221.2009.00725.x

[7] Mayda A.M, Rodrik D., (2005). Why are some people (and countries) more protectionist than others? *European Economic Review*, 49(6), 1393–1430. doi: 10.1016/j.euroecorev.2004.01.002

[8] Slemrod J., (2007). Cheating ourselves: the economics of tax evasion. *Journal of Economic Perspectives*, 21(1), 25–48. doi: 10.1257/jep.21.1.2519728420

[9] Van Hilvoorde I., Elling A. Stokvis R., (2010). How to influence national pride? The Olympic medal index as a unifying narrative. *International Review for the Sociology of Sport*, 45(1), 87–102. doi: 10.1177/1012690209356989

[10] Kim Y., KiTae Y., Ko Y.J., (2013). Consumer patriotism and response to patriotic advertising: Comparison of international vs. national sport events. *International Journal of Sports Marketing and Sponsorship*, 14(3), 229–251. doi: 10.1108/IJSMS-14-03-2013-B006

[11] Clift B., Woll C., (2012). Economic patriotism: reinventing control over open markets. *Journal of European Public Policy*, 19(3), 307–323. doi: 10.1080/13501763.2011.638117

[12] Mercer P., (2009). Australians go on a spending spree. *The National*. June 9, 2009. Available at: https://www.thenationalnews.com/world/asia/australia-goes-on-a-spending-spree-1.592930

[13] Risse L., (2010). ‘… And one for the country’ The effect of the baby bonus on Australian women’s childbearing intentions. *Journal of Population Research*, 27(3), 213–240. doi: 10.1007/s12546-011-9055-4

[14] Hertzberg H., 2011. Off and Running. The New Yorker. May 23, (2011). Available at: https://www.newyorker.com/magazine/2011/05/23/off-and-running-hendrik-hertzberg.

[15] Torgler B., (2004). Tax morale in Asian countries. *Journal of Asian Economics*, 15(2), 237–266. doi: 10.1016/j.asieco.2004.02.001

[16] Torgler B., (2005). Tax morale in Latin America. *Public Choice*, 122(1–2), 133–157. doi: 10.1007/s11127-005-5790-4

[17] Torgler B., (2007). *Tax compliance and tax morale*: *A theoretical and empirical analysis*, Edward Elgar Publishing.

[18] Wenzel M., (2007). The multiplicity of taxpayer identities and their implications for tax ethics. *Law & Policy*, 29(1), 31–50. doi: 10.1111/j.1467-9930.2007.00244.x

[19] Gangl K., Torgler B., Kirchler E., (2016). Patriotism’s impact on cooperation with the state: an experimental study on tax compliance. *Political Psychology*, 37(6), 867–881. doi: 10.1111/pops.12294 27980350 PMC5125400

[20] Dulleck U., Fooken J., Newton C., Ristl A., Schaffner M., Torgler B., (2016). Tax compliance and psychic costs: Behavioral experimental evidence using a physiological marker. *Journal of Public Economics*, 134, 9–18. doi: 10.1016/j.jpubeco.2015.12.007

[21] Dulleck U., Schaffner M., Torgler B. (2014). Heartbeat and economic decisions: observing mental stress among proposers and responders in the ultimatum bargaining game. *PLoS One*, 9(9), e108218. doi: 10.1371/journal.pone.0108218 25247817 PMC4172752

[22] Alm J., (1999). Tax compliance and administration. In: Hildreth W.B., Richardson J.A. (Eds.), *Handbook on Taxation*. Marcel Dekker, New York, pp. 741–768.

[23] Alm J., (2019). What motivates tax compliance?. *Journal of Economic Surveys*, 33(2), 353–388. doi: 10.1111/joes.12272

[24] Torgler B., (2002). Speaking to theorists and searching for facts: Tax morale and tax compliance in experiments. *Journal of Economic Surveys*, 16(5), 657–683. doi: 10.1111/1467-6419.00185

[25] Alm J., Malézieux A., (2020). 40 years of tax evasion games: A meta-analysis, *Experimental Economics*, 24, 699–750. doi: 10.1007/s10683-020-09679-3

[26] Greiner B., (2004). *The Online Recruitment System ORSEE 2*.*0—A Guide for the Organization of Experiments in Economics*, Technical report, University of Cologne, Department of Economics.

[27] Schaffner M., (2013). *Programming for experimental economics*: *Introducing CORAL—a lightweight framework for experimental economic experiments*. Technical report, QUT Business School.

[28] Durham Y., Manly T.S., Ritsema C., (2014). The effects of income source, context, and income level on tax compliance decisions in a dynamic experiment. *Journal of Economic Psychology*, 40, 220–233. doi: 10.1016/j.joep.2012.09.012

[29] Bellemare C., Deversi M., Englmaier F., (2019). Complexity and distributive fairness interact in affecting compliance behavior. *CESifo Working Paper No*. *7899*, Available at SSRN. 10.2139/ssrn.3474206

[30] Hartner-Tiefenthaler M., Rechberger S., Kirchler E., (2013). Justice perceptions and cooperation of citizens with the tax-authorities: The group engagement model of cooperation. *Citizenship Teaching & Learning*, 8(2), 179–193. doi: 10.1386/ctl.8.2.179_1

[31] Dulleck U., Ristl A., Schaffner M., Torgler B., (2011). Heart Rate Variability, the Autonomous Nervous System, and Neuroeconomic Experiments. *Journal of Neuroscience*, *Psychology*, *and Economics*, 4(2), 117–124. https://psycnet.apa.org/doi/10.1037/a0022245.

[32] Torgler B., (2019). Opportunities and challenges of portable biological, social, and behavioral sensing systems for the social sciences’, In: Foster G. (Ed.), *Biophysical Measurement in Experimental Social Science Research*: *Theory and Practice*. Academic Press, Elsevier, pp. 197–224.

[33] Appelhans B.M., Luecken L. J., (2006). Heart rate variability as an index of regulated emotional responding. *Review of General Psychology*, 10(3), 229–240. doi: 10.1037/1089-2680.10.3.229

[34] Falk A., Menrath I., Verde P.E, Siegrist J., (2011). *Cardiovascular consequences of unfair pay*, Technical report. Discussion paper series. Forschungsinstitut zur Zukunft der Arbeit.

[35] Porges S.W., (2011). *The Polyvagal Theory*: *Neurophysiological Foundations of Emotions*, *Attachment*, *Communication*, *and Self-regulation* (Norton Series on Interpersonal Neurobiology), WW Norton & Company.

[36] Porges S.W., (2007a). A phylogenetic journey through the vague and ambiguous Xth cranial nerve: A commentary on contemporary heart rate variability research. *Biological Psychology*, 74(2), 301–307. doi: 10.1016/j.biopsycho.2006.08.007 17055142 PMC1828879

[37] Porges S.W., (2007b). The polyvagal perspective. *Biological Psychology*, 74(2), 116–143. doi: 10.1016/j.biopsycho.2006.06.009 17049418 PMC1868418

[38] Djawadi B. M., Fahr R. (2013). *The impact of tax knowledge and budget spending influence on tax compliance* (No. 7255). IZA Discussion Papers.

[39] Uğraş G. A., Yıldırım G., Yüksel S., Öztürkçü Y., Kuzdere M., Öztekin S.D., (2018). The effect of different types of music on patients’ preoperative anxiety: A randomized controlled trial. Complement Ther. *Clinical Practice*, 31, 158–163. doi: 10.1016/j.ctcp.2018.02.012 29705448

[40] Burns J., Labb E., Williams K., McCall J., (1999). Perceived and physiological indicators of relaxation: as different as Mozart and Alice in Chains. *Applied Psychophysiology and Biofeedback*, 24(3), 197–202. doi: 10.1023/a:1023488614364 10652638

[41] Chabris C.F., (1999). Prelude or requiem for the ‘Mozart effect’? *Nature*, 400(6747), 826–827. doi: 10.1038/23608 10476958

[42] Braithwaite V., (2017). Dancing with tax authorities: Motivational postures and non-compliant actions. In: Braithwaite V. (Ed.), *Taxing Democracy*: *Understanding Tax Avoidance and Evasion*. Routledge, pp. 15–40.

[43] Taylor N., McGarty C., (2001). The role of subjective group memberships and perceptions of power in industrial conflict. *Journal of Community &* *Applied Social Psychology*, 11(5), 389–93. doi: 10.1002/casp.636

[44] Becker J.C., Enders-Comberg A., Wagner U., Christ O., Butz D.A., (2012). Beware of national symbols. *Social Psychology*, 43(1), 3–6. doi: 10.1027/1864-9335/a000073

[45] Chan H.F., Dulleck U., Fooken J., Moy N., Torgler B., (2022). Cash and the hidden economy: experimental evidence on fighting tax evasion in small business transactions. *Journal of Business Ethics*, doi: 10.1007/s10551-022-05186-y

[46] Torgler B., (2013). A field experiment in moral suasion and tax compliance focusing on underdeclaration and overdeduction. *FinanzArchiv*: *Public Finance Analysis*, 69, 393–411.

[47] Torgler B. Schneider F., (2007). What shapes attitudes toward paying taxes? Evidence from multicultural European countries. *Social Science Quarterly*, 88(2), 443–470. doi: 10.1111/j.1540-6237.2007.00466.x

