# Supplementary figures and images for: National pride and tax compliance: A laboratory experiment using a physiological marker
Source: PLoS One. 2023 Jan 19;18(1):e0280473. doi: 10.1371/journal.pone.0280473 (PMC9851525; doi:10.1371/journal.pone.0280473)

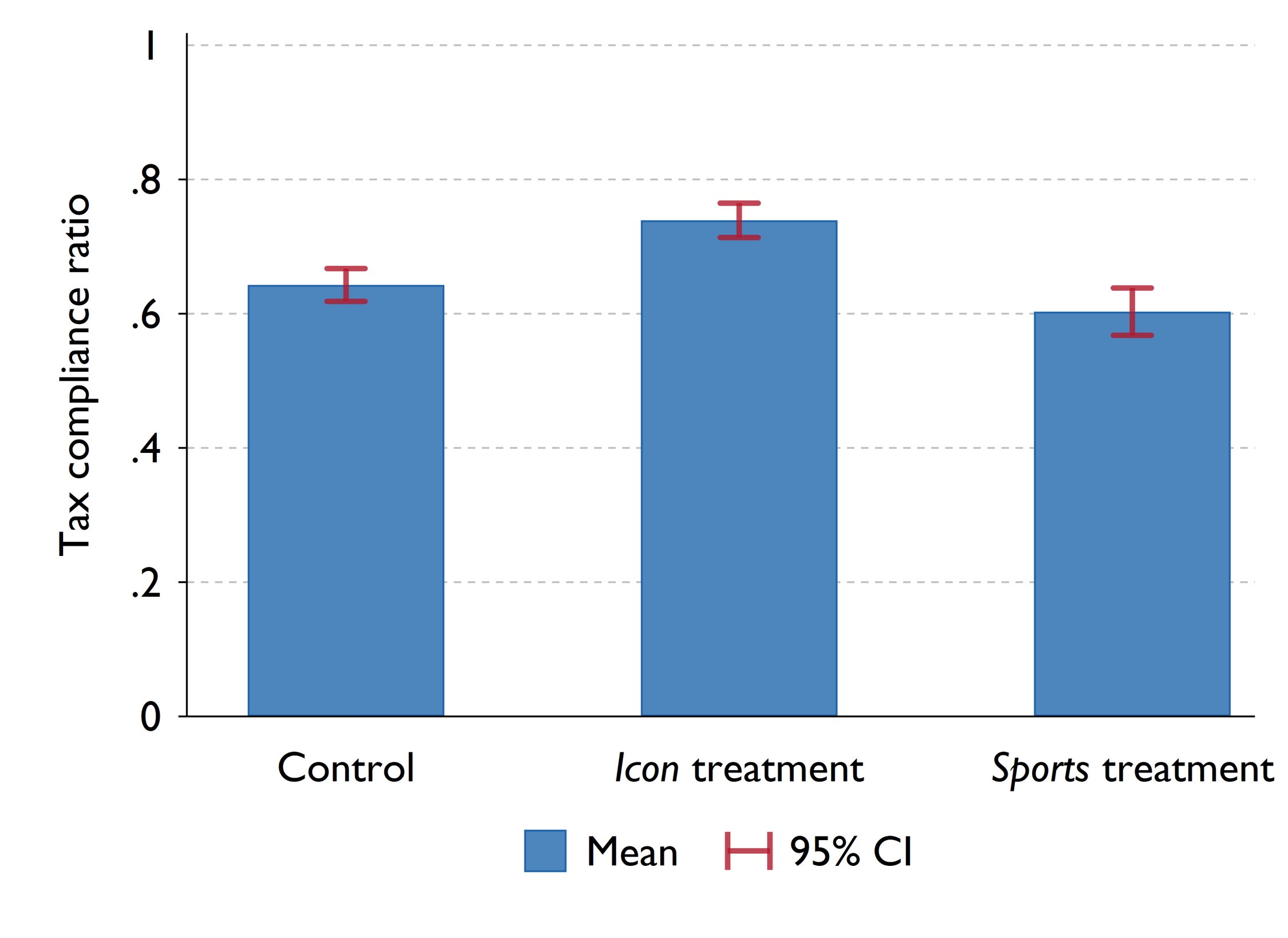

Supplement: S1 Fig — Effect of treatment on average tax compliance ratio. Participants in the treatment using iconic images to trigger national pride demonstrated a higher level of compliance relative to those in the control. Error bars represent 95% confidence intervals. (TIF) [file pone.0280473.s001.tif]

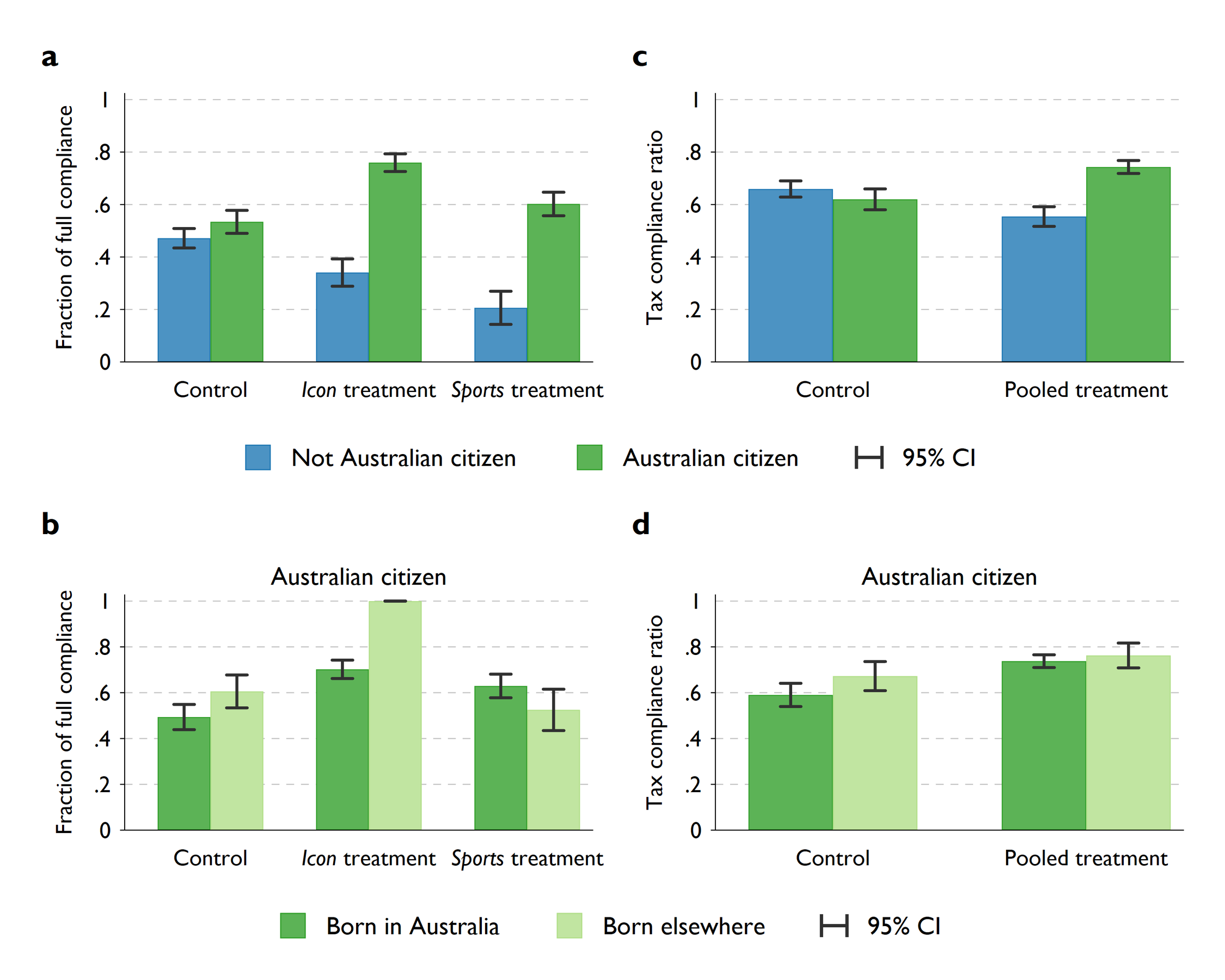

Supplement: S2 Fig — Panels a and b show the average fraction of full tax compliance (extensive margins of tax compliance) by treatment group (control, icon, and sport) and participants’ citizenship status. Icon and sport treatments are pooled in panels c and d, which show average tax compliance ratio for participants in the respective group. Panels b and d restricts the sample to Australians participants. Error bars represent 95% confidence intervals. (TIF) [file pone.0280473.s002.tif]

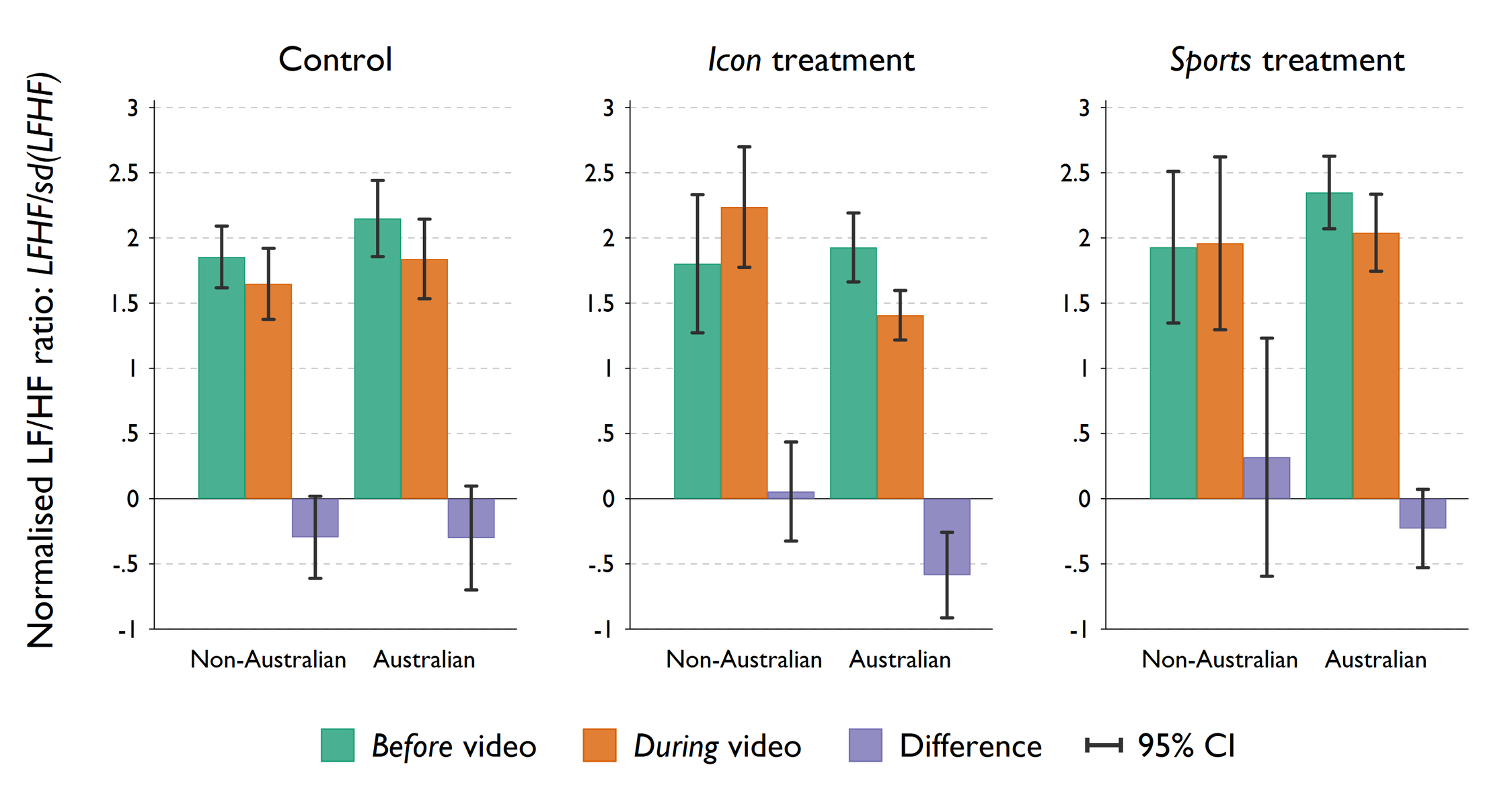

Supplement: S3 Fig — The figure shows the median normalised LF/HF ratio (stress indicator) before and during the video framing and the corresponding difference. Error bars represent 95% confidence intervals. (TIF) [file pone.0280473.s003.tif]

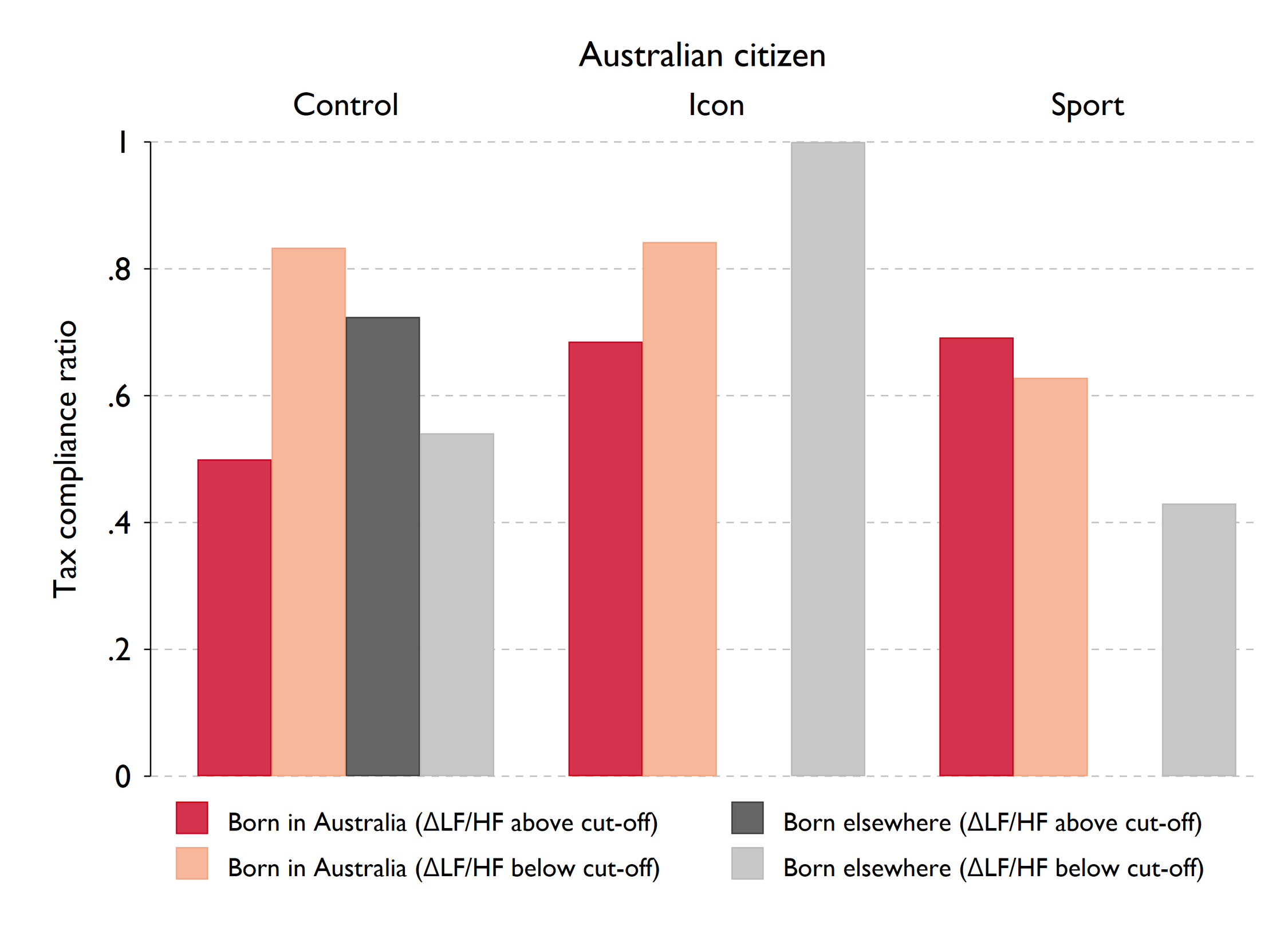

Supplement: S4 Fig — The figure shows the average level of tax compliance of Australian participants by their country of birth (red bars represent participants were born in Australia and grey bars represent participants who were born elsewhere). Caution should be exercised when interpreting the results due to the small number of foreign-born Australian participants. (TIF) [file pone.0280473.s004.tif]

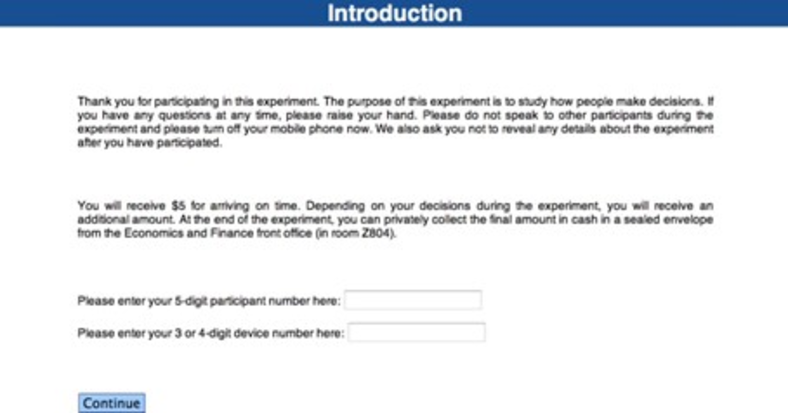

Supplement: S5 Fig — Screenshot of introduction screen. (TIF) [file pone.0280473.s005.tif]

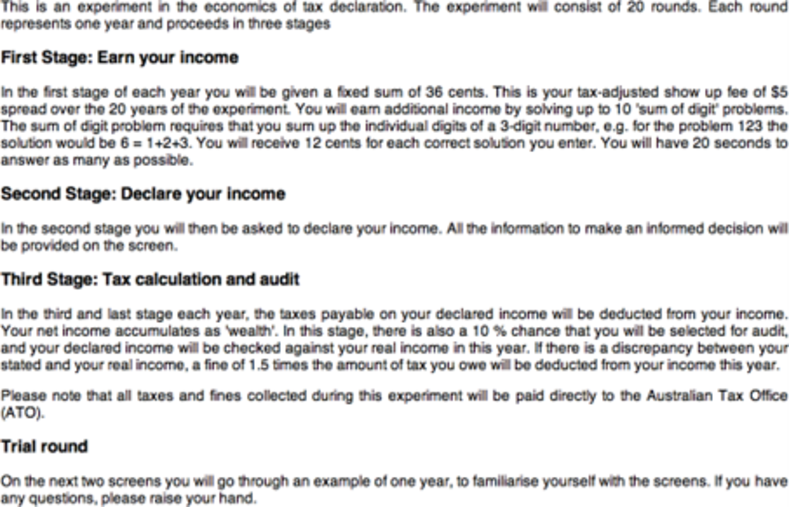

Supplement: S6 Fig — Screenshot of instruction screen. (TIF) [file pone.0280473.s006.tif]

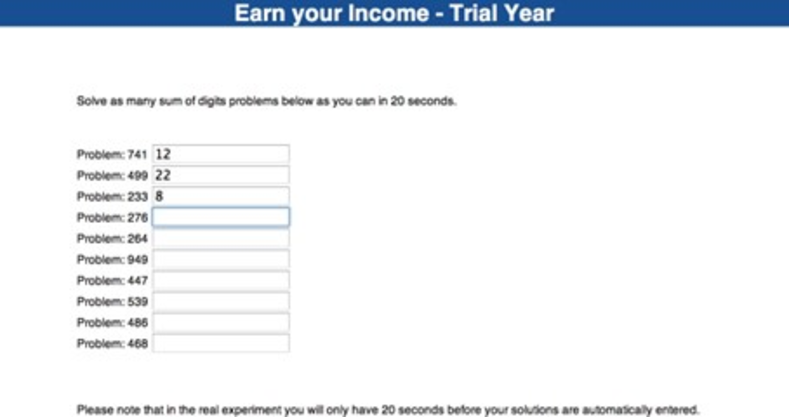

Supplement: S7 Fig — Screenshot of earn income trial screen. (TIF) [file pone.0280473.s007.tif]

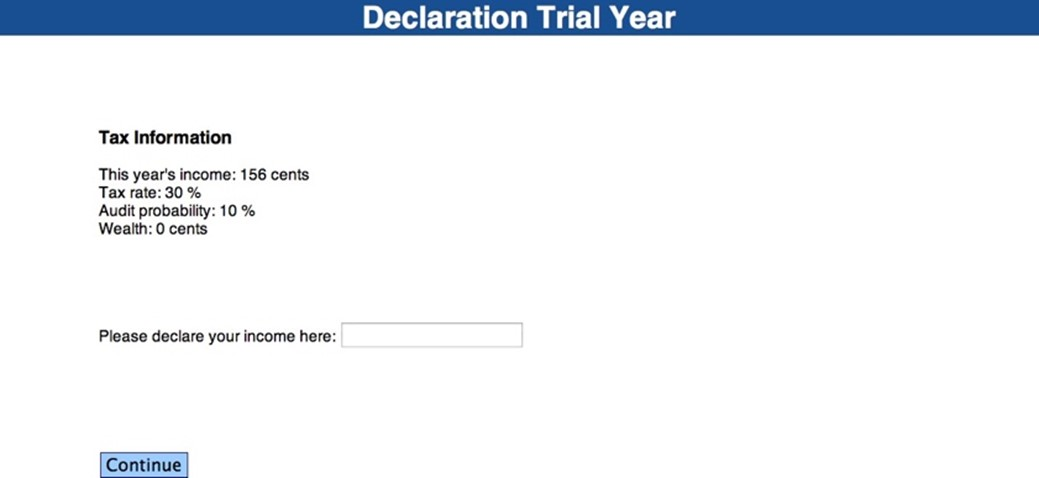

Supplement: S8 Fig — Screenshot of trial declaration screen. (TIF) [file pone.0280473.s008.tif]

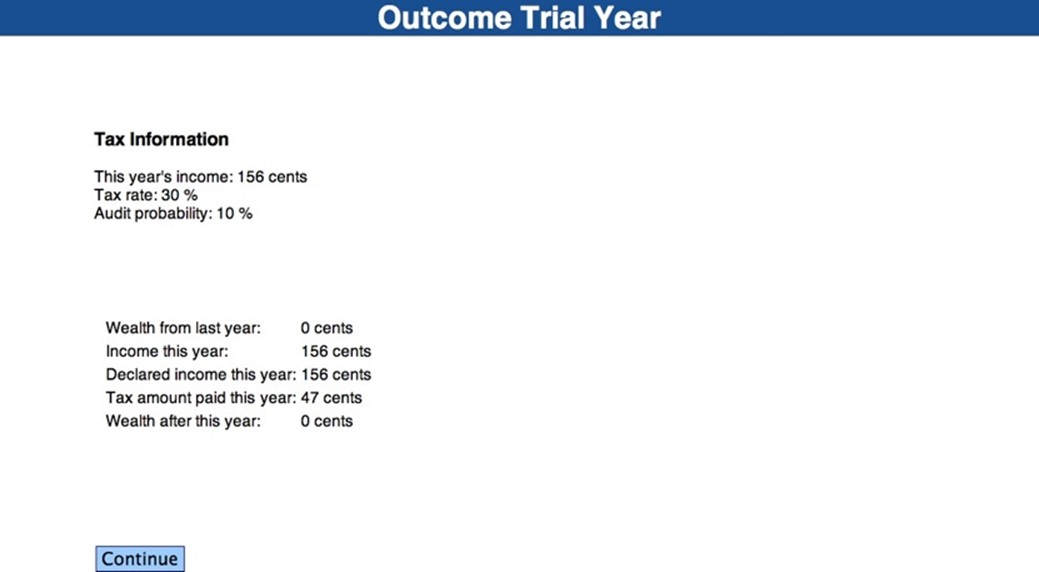

Supplement: S9 Fig — Screenshot of outcome screen. (TIF) [file pone.0280473.s009.tif]

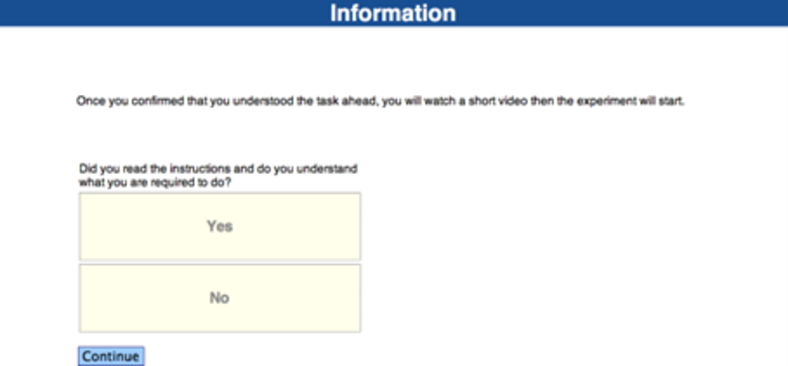

Supplement: S10 Fig — Screenshot of understand instructions screen. (TIF) [file pone.0280473.s010.tif]

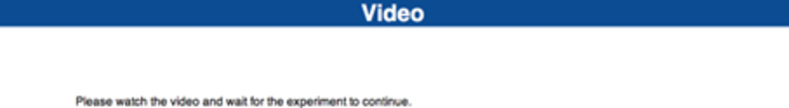

Supplement: S11 Fig — Screenshot of waiting screen before video started. (TIF) [file pone.0280473.s011.tif]

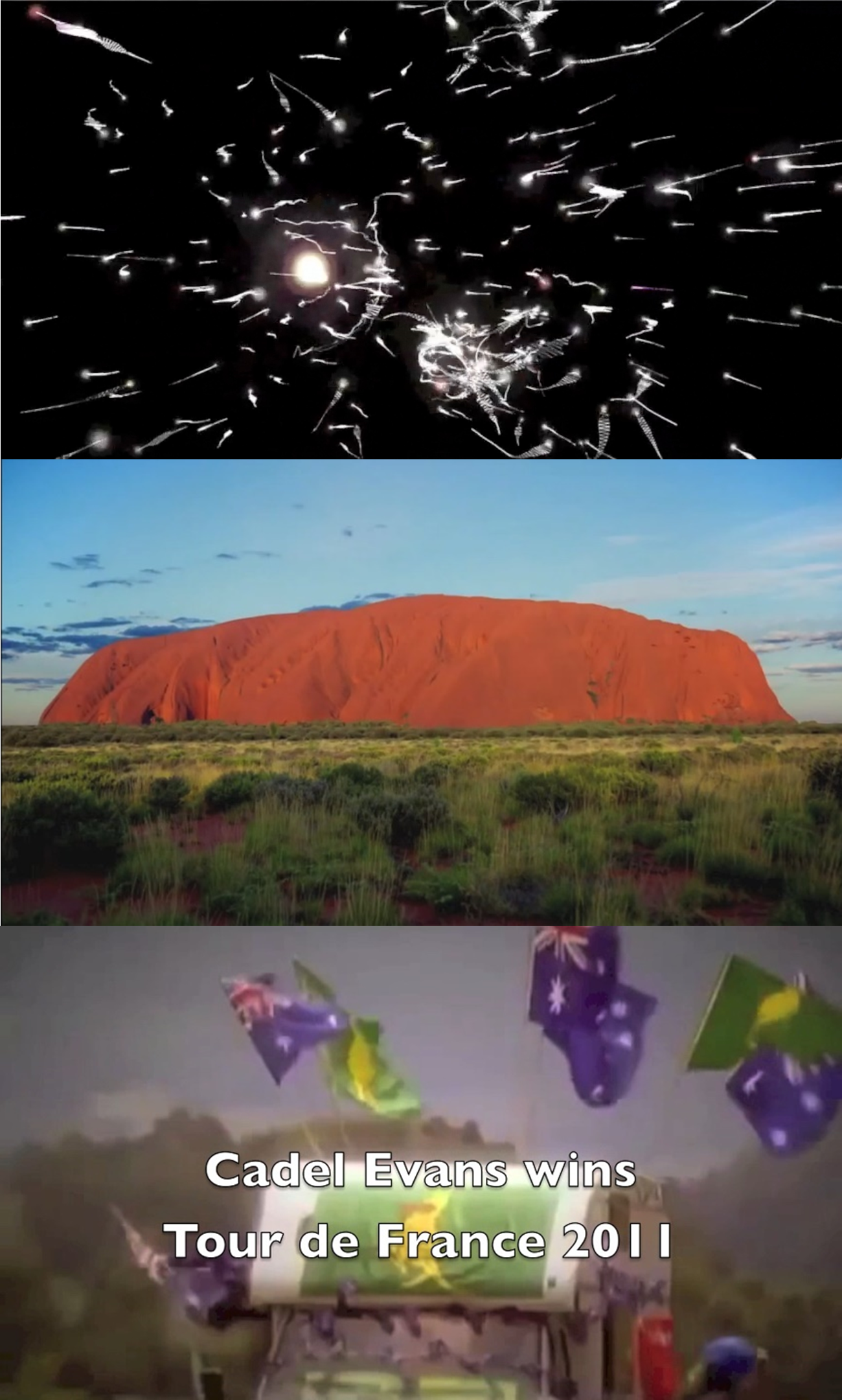

Supplement: S12 Fig — Top panel (control video)–screenshot of visualisation generated by iTunes. Middle and bottom panels show a screenshot from the icon and sport treatment video, respectively. (TIF) [file pone.0280473.s012.tif]

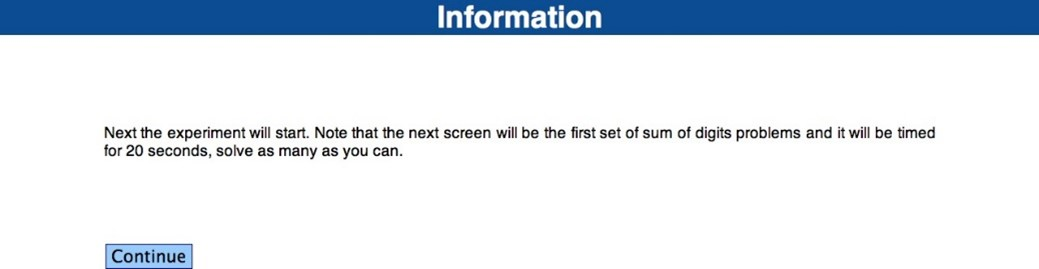

Supplement: S13 Fig — Screenshot of post-video information screen. (TIF) [file pone.0280473.s013.tif]

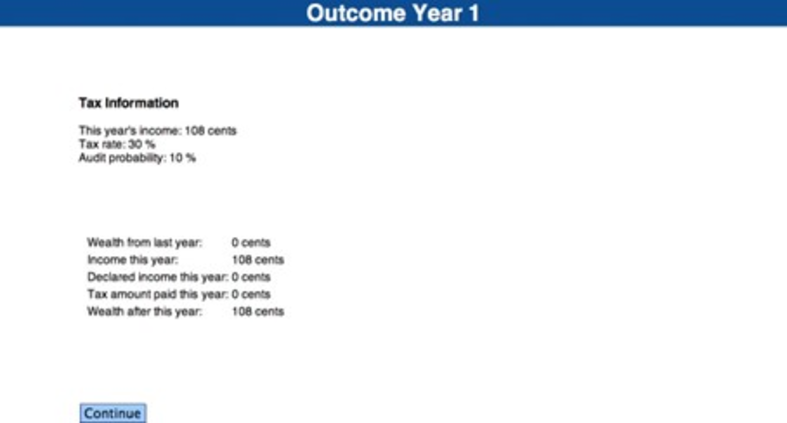

Supplement: S14 Fig — Screenshot of full defection screen. (TIF) [file pone.0280473.s014.tif]

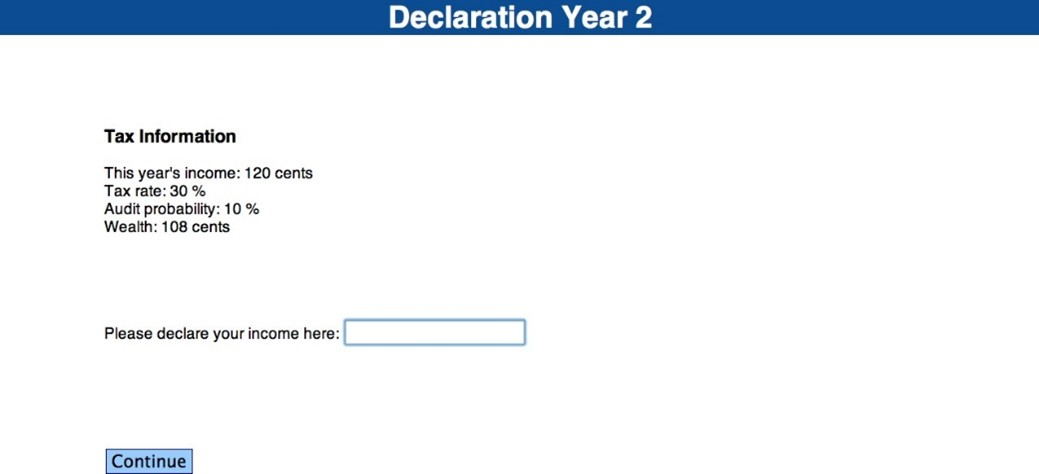

Supplement: S15 Fig — Screenshot of declaration screen. (TIF) [file pone.0280473.s015.tif]

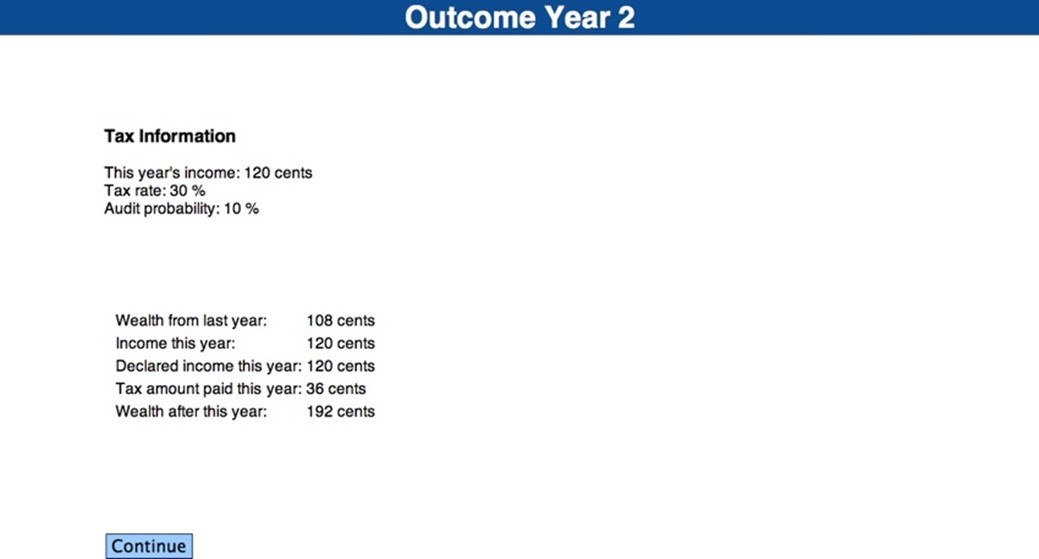

Supplement: S16 Fig — Screenshot of full compliance screen. (TIF) [file pone.0280473.s016.tif]

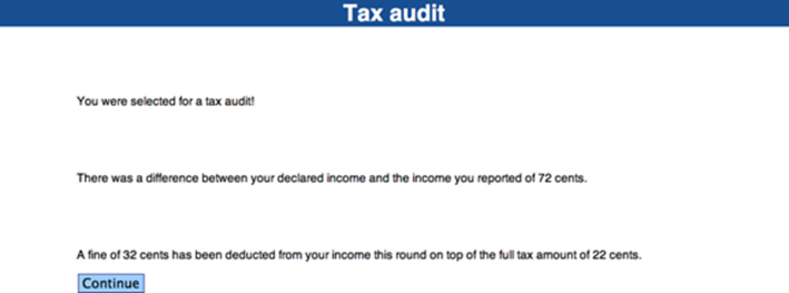

Supplement: S17 Fig — Screenshot of audit outcome. (TIF) [file pone.0280473.s017.tif]

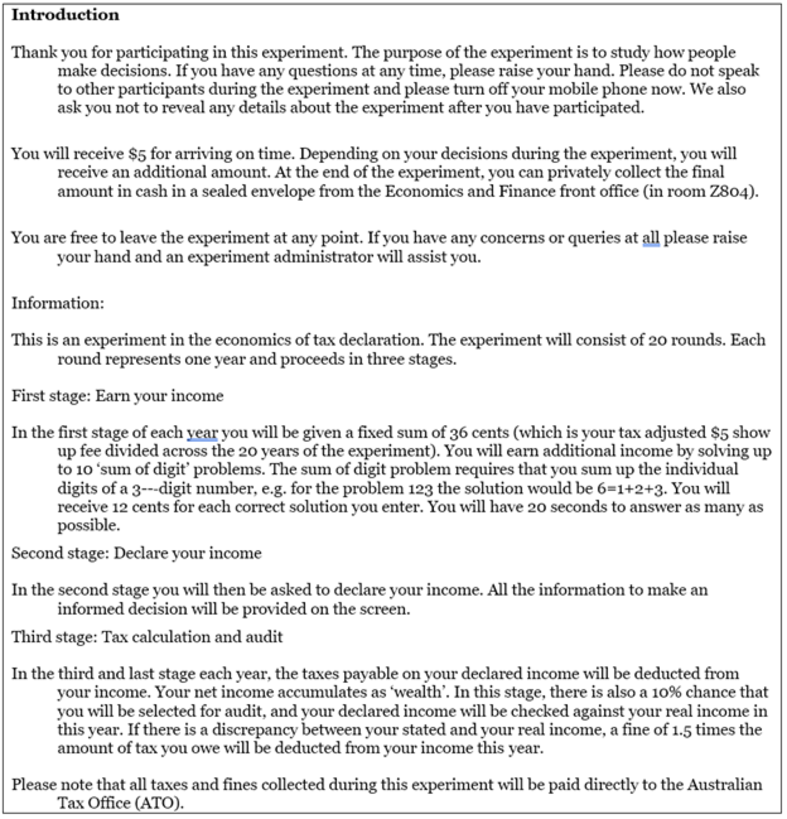

Supplement: S18 Fig — ATO version. (TIF) [file pone.0280473.s018.tif]

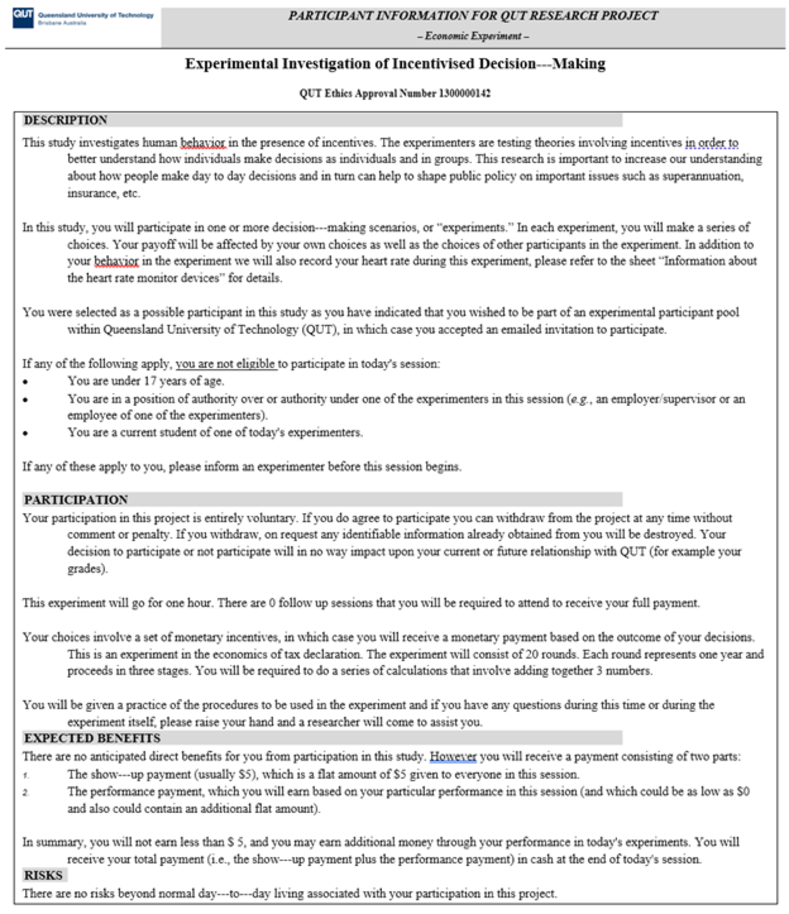

Supplement: S19 Fig — (TIF) [file pone.0280473.s019.tif]

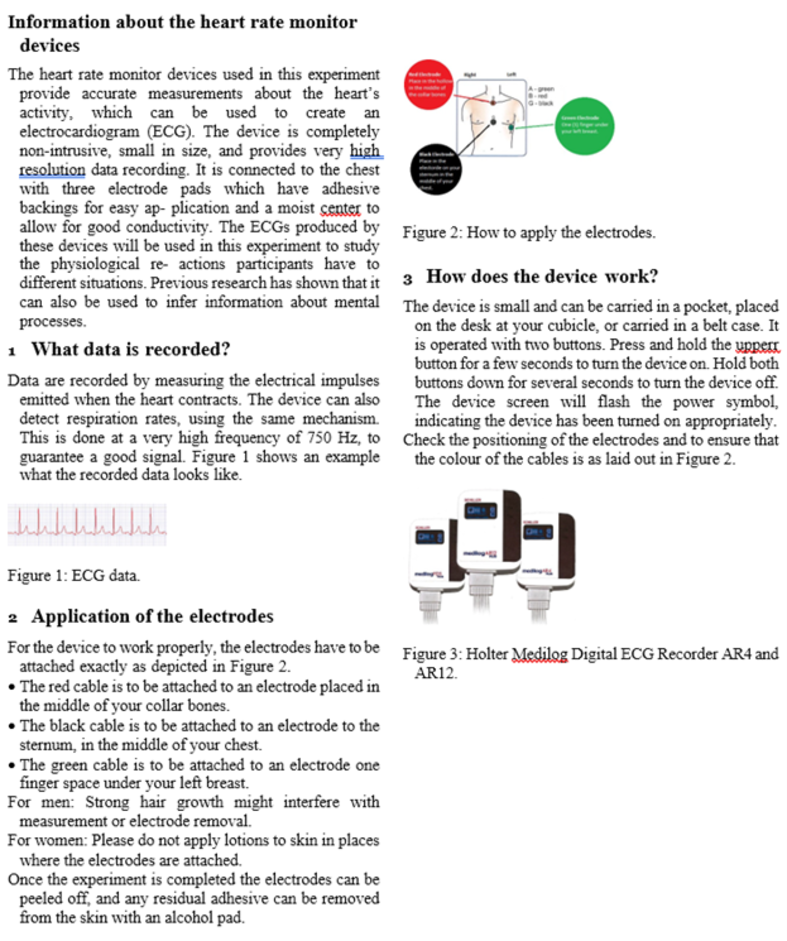

Supplement: S20 Fig — Instructions on attaching HRV monitor. (TIF) [file pone.0280473.s020.tif]

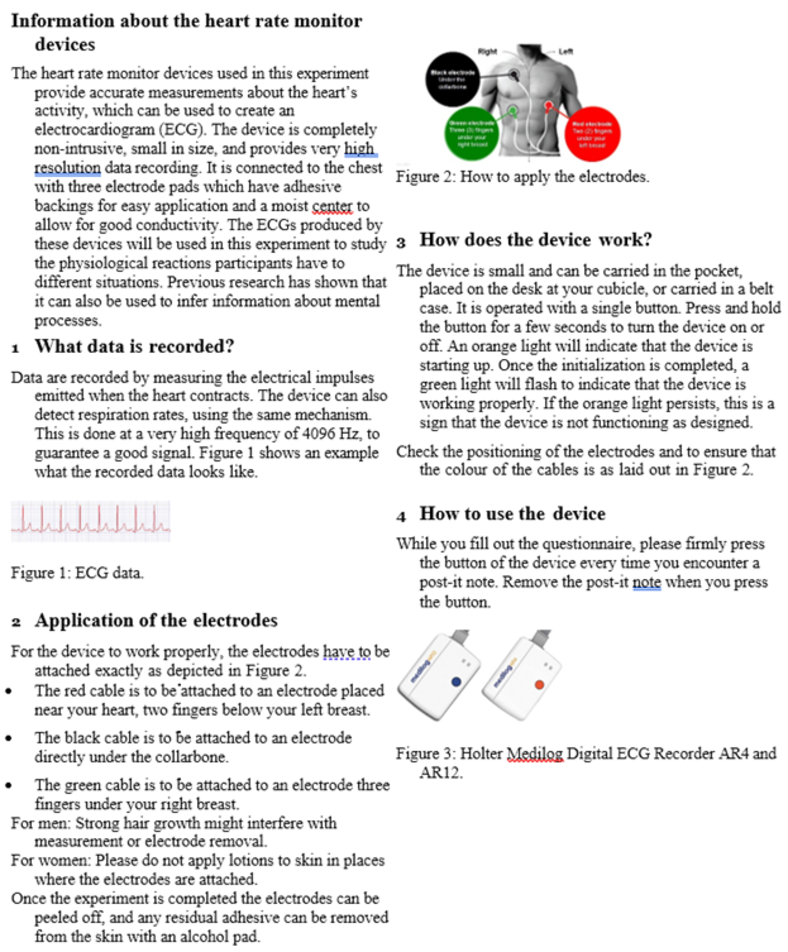

Supplement: S21 Fig — Instructions on attaching HRV monitor. (TIF) [file pone.0280473.s021.tif]

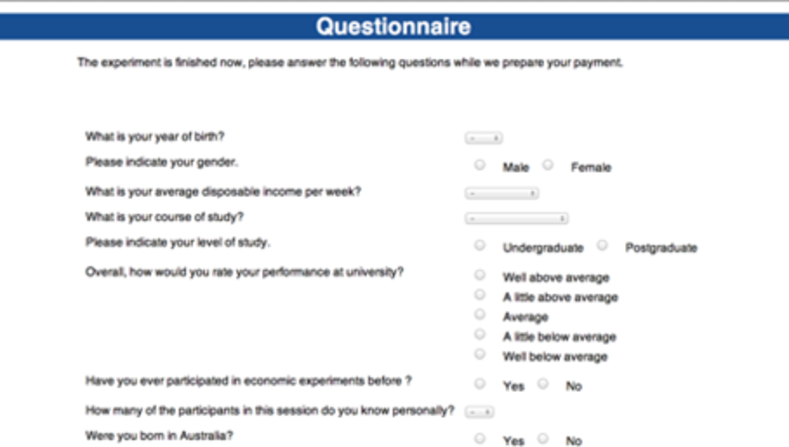

Supplement: S22 Fig — (TIF) [file pone.0280473.s022.tif]

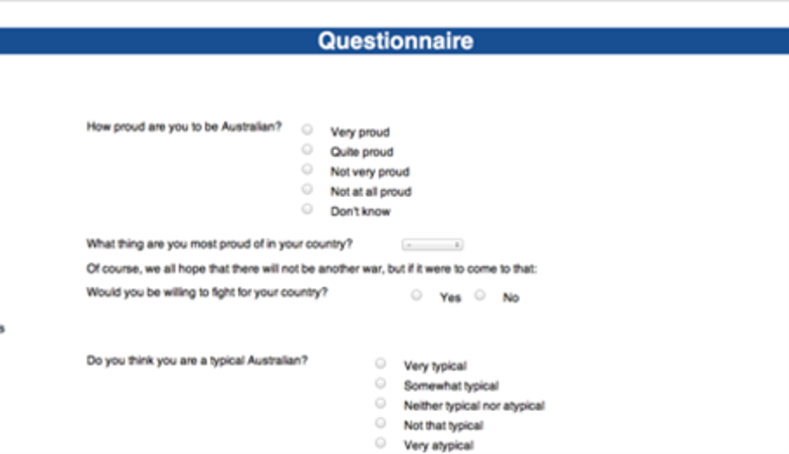

Supplement: S23 Fig — First screen of questionnaire for those born in Australia. (TIF) [file pone.0280473.s023.tif]

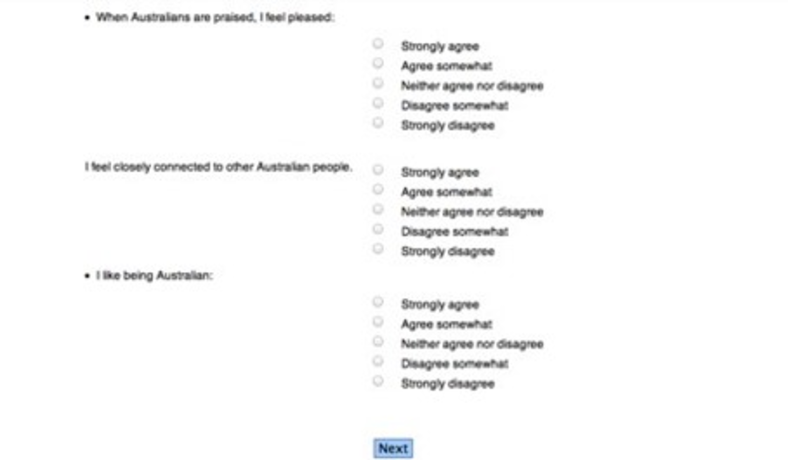

Supplement: S24 Fig — Second screen of questionnaire for those born in Australia. (TIF) [file pone.0280473.s024.tif]

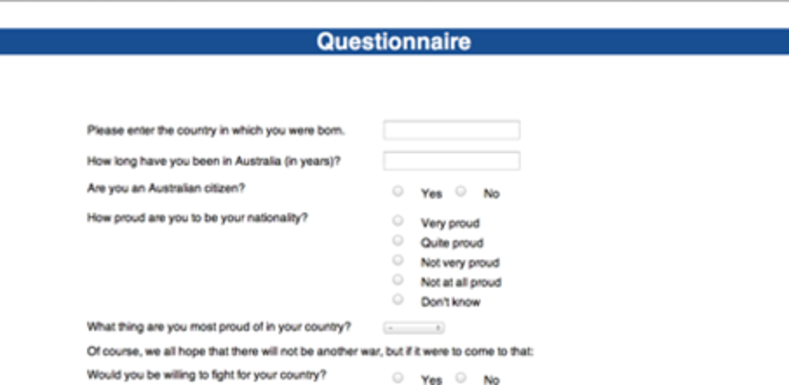

Supplement: S25 Fig — First screen of questionnaire for those born overseas. (TIF) [file pone.0280473.s025.tif]

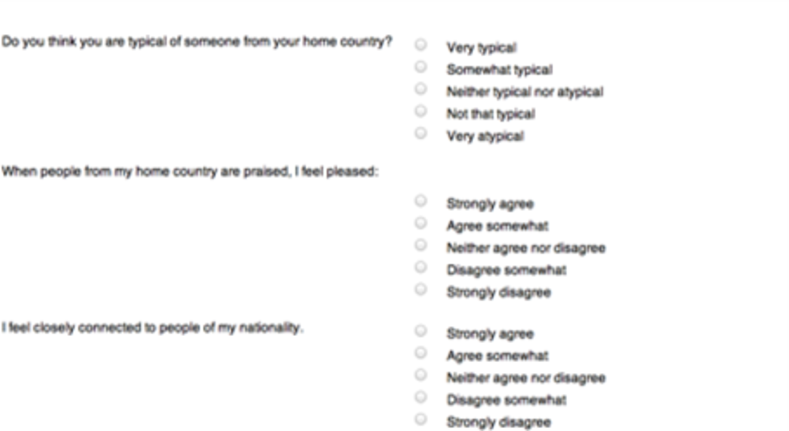

Supplement: S26 Fig — Second screen of questionnaire for those born overseas. (TIF) [file pone.0280473.s026.tif]

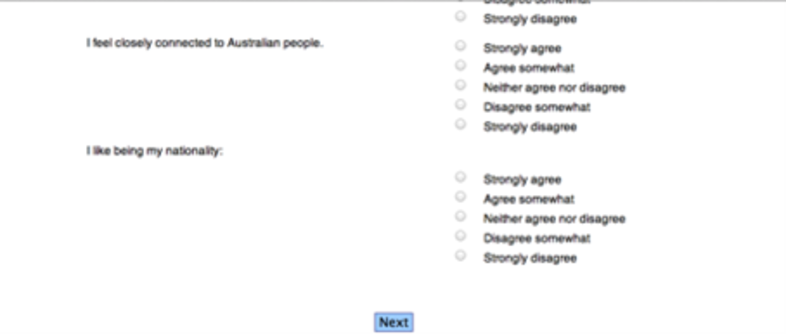

Supplement: S27 Fig — Third screen of questionnaire for those born overseas. (TIF) [file pone.0280473.s027.tif]

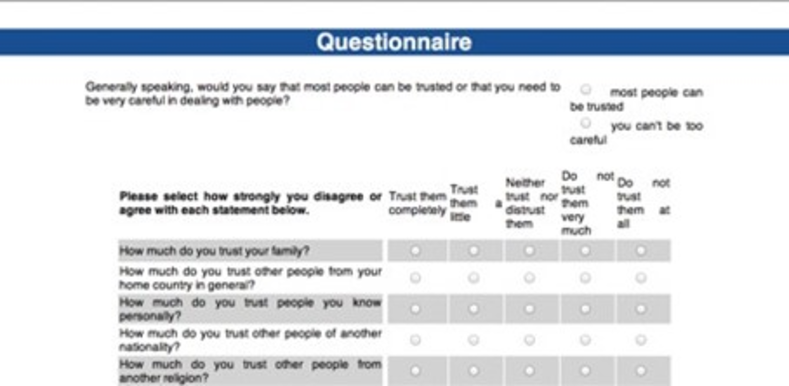

Supplement: S28 Fig — Second screen of questionnaire for all. (TIF) [file pone.0280473.s028.tif]

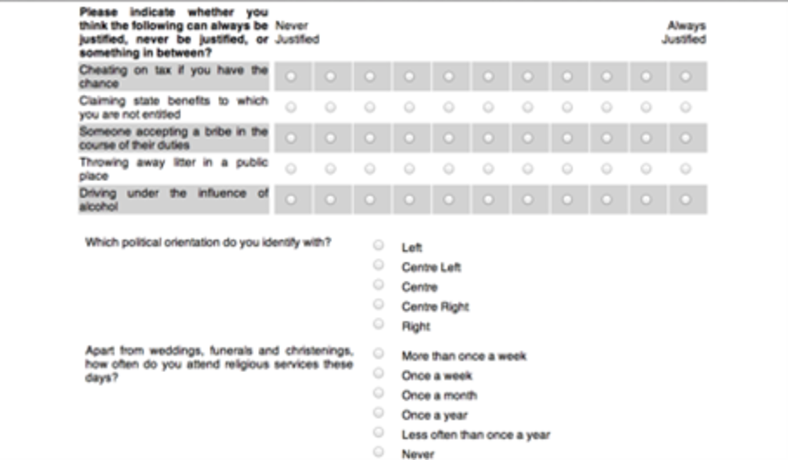

Supplement: S29 Fig — Third screen of questionnaire for all. (TIF) [file pone.0280473.s029.tif]

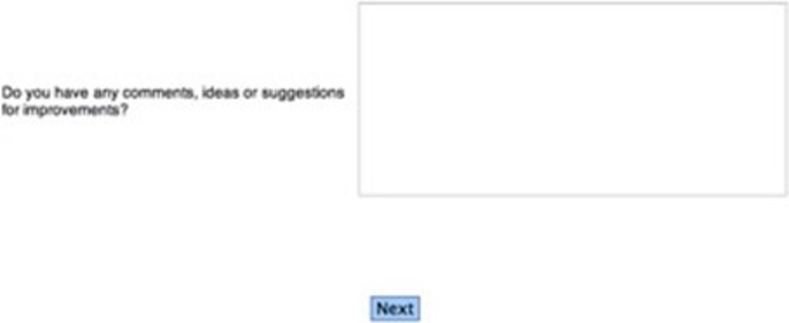

Supplement: S30 Fig — Fourth screen of questionnaire for all. (TIF) [file pone.0280473.s030.tif]

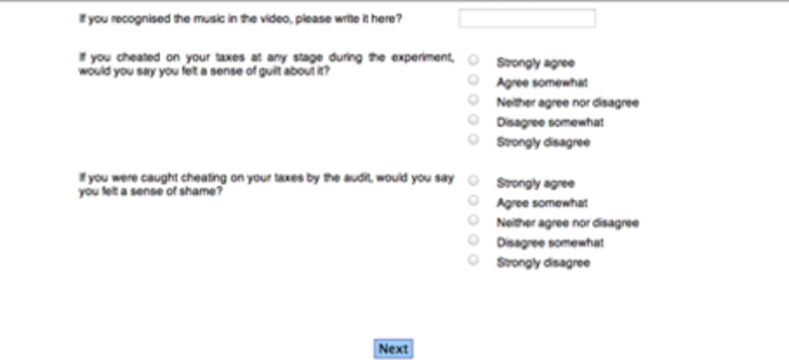

Supplement: S31 Fig — Fifth screen of questionnaire for all. (TIF) [file pone.0280473.s031.tif]

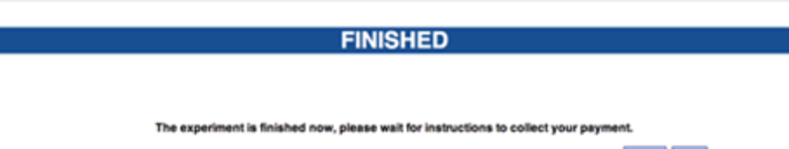

Supplement: S32 Fig — Final screen of questionnaire for all. (TIF) [file pone.0280473.s032.tif]
